# Spin-Flip TDDFT within the Sternheimer Formulation: A Gaussian and Plane Wave Implementation

**DOI:** 10.1021/acs.jpca.5c05234

**Published:** 2025-10-14

**Authors:** Luis I. Hernandez-Segura, Sandra Luber

**Affiliations:** Department of Chemistry, 27217 University of Zürich, 8057 Zürich, Switzerland

## Abstract

We report the first implementation of spin-flip time-dependent density functional theory (SF-TDDFT) within the Tamm-Dancoff approximation in the Sternheimer formulation including the use of the noncollinear kernel. The noncollinear kernel was stabilized by introducing a screening method for the numerical integration, realizing a robust scheme of excited energy and gradient calculations of SF-TDDFT using generalized gradient approximation functionals. The implementation is evaluated by benchmark calculations of vertical excitation energies and optimized molecular geometries. The benchmark for vertical excitations consists of 19 excitations with high level of theory reference data from the QUESTDB. An underestimation of vertical excitation energies was observed for the PBE and PBE0 functionals, as seen by their average deviations of −0.3 eV. The benchmark for optimized geometries consists of 25 optimized structures with high level of theory, comprising CCSD, CISD, and FCI data, and 10 reference structures optimized with other implementations of collinear and noncollinear SF-TDDFT. The optimized structures using PBE and PBE0, with a noncollinear kernel, were found to be close to the high-level reference structures, with mean deviations of 0.010 and −0.004 Å, respectively. The extension to the auxiliary density matrix method (ADMM) is also presented. We found an average deviation of 0.003 Å in the calculated bond lengths when employing the ADMM for the PBE0 functional.

## Graphical abstract

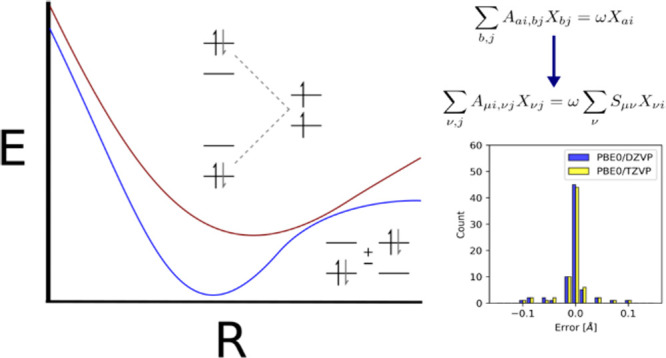

## Introduction

Linear response time-dependent density functional theory (TDDFT)^1^ is one of the most widely used methods for calculating excited-state properties. With an accuracy of approximately 0.3 eV for vertical excitation energies, compared to theoretical best estimates, and a computational scaling below cubic, TDDFT offers a good balance between accuracy and computational cost.^2,3^ However, it is not without its flaws. A major issue is its inability to accurately describe multireference closed-shell singlet states. These states can only be captured, within the framework of single excitations, through spin-flip excitations. Taking account of multiple closed-shell states is particularly crucial when studying molecular geometries where the ground state’s potential energy surface becomes near-degenerate with that of an excited state, for example, during chemical bond formation and breaking, or in systems with diradical character.^4,5^

One way to address this limitation is by using spin-flip TDDFT (SF-TDDFT). In this approach, the lowest triplet state is first calculated using self-consistent field (SCF) methods, and the true singlet ground state with multireference character is obtained through spin-flip excitations. There are two main types of SF-TDDFT: collinear^5^ and noncollinear.^6^ The distinction between them lies in the exchange-correlation kernel. In collinear SF-TDDFT, the contribution from the local or semilocal exchange-correlation functional is neglected, whereas in noncollinear SF-TDDFT, a noncollinear kernel is employed to account for these effects.

Noncollinear SF-TDDFT offers more accurate excitation energies compared to its collinear counterpart and has been generally successful when applied with local density approximation (LDA) functionals.^7^ However, the noncollinear kernel is ill-defined for generalized gradient approximation (GGA) functionals, leading to singularities during numerical integration. These singularities not only impact the calculated energies but also exacerbate errors in their derivatives.^8^ Addressing these issues remains an active area of research^9–16^ and falls outside the scope of this work. Instead of tackling the noncollinear kernel problem directly for GGAs, we focus on stabilizing the numerical integration for noncollinear SF-TDDFT^6^ and developing the corresponding analytic energy gradient calculations. Our approach is based on the expectation that a robust solution to the noncollinear kernel problem will emerge in the near future, allowing for its seamless integration into the framework.

Although the accuracy and computational efficiency of SF-TDDFT is comparable to standard TDDFT,^7^ its reliance on explicit unoccupied molecular orbitals (MOs) presents a challenge for large systems, as it requires full diagonalization of the Kohn–Sham matrix. This limitation can be addressed by reformulating SF-TDDFT within the Sternheimer perturbation theory,^17^ similar to previous work on TDDFT.^18,19^ In this approach, the unoccupied space is implicitly handled by using projector matrices that project onto the occupied space, needing only a few unoccupied MOs for the building of the initial guess for the Davidson solver used to iteratively calculate the excitation energies.

A key advantage of the Sternheimer formulation is that most matrix operations are performed in an atomic orbital basis, allowing the use of the Distributed Block Compressed Sparse Row (DBCSR) library.^20^ This highly efficient and parallelized library is optimized to take full advantage of matrix sparsity, while still being effective for dense matrices.

Additionally, the implementation in CP2K employs the Gaussian and Plane Waves (GPW) method.^21^ This approach enables efficient evaluation of Coulomb, exchange and correlation terms by allowing the electronic density to be represented using Gaussian-type atomic orbitals, or plane waves through a Fourier transform.

In SF-TDDFT calculations, the inclusion of Fock exchange is mandatory for collinear SF-TDDFT.^5^ The computation of Fock integrals formally scales with the fourth power of the system size, which can limit the feasibility of large-scale applications. This issue can be alleviated by employing the auxiliary density matrix method (ADMM).^22,23^ In ADMM, Fock exchange contributions are approximated using a smaller auxiliary basis set, with a correction term based on a local functional to minimize the loss of accuracy. The resulting errors are typically small; around 0.2 pm for bond lengths of optimized excited-state geometries and 0.02 eV for excitation energies when using Triple-ζ basis sets.^19^

Although the formal scaling of the Fock exchange is still fourth order, it can be reduced to below cubic by employing standard screening techniques. Notably, this reduced scaling applies to the smaller auxiliary basis set, leading to Fock exchange computation times comparable to those for the exchange-correlation term.^19,23^

In the following section, we introduce a Lagrangian formulation in the Sternheimer formalism for spin-flip excitation energies that eliminates the need for unoccupied molecular orbitals. We extend this Lagrangian to incorporate the Z-vector method, which is crucial for the calculation of analytic derivatives. Additionally, we present the necessary modifications to the equations when employing the ADMM. Lastly, we validate the accuracy of the excitation energies, optimized structures, and the performance of the ADMM.

## Theoretical Methods

### Theory

The notation in this work closely follows the TDDFT implementation in CP2K.^19^ Indices *i*, *j*, *k*, ··· refer to occupied MOs, while *a*, *b*, *c*, ··· denote unoccupied MOs. Arbitrary MOs are labeled using *p*, *q*, *r*, ···. For atomic orbitals (AOs), we use μ, ν, τ, η. Spin-up and spin-down are labeled as α and β, respectively, while an arbitrary spin is denoted as σ. The set of all occupied and unoccupied MOs are labeled as *occ* and *uno*, respectively. All four-center integrals are expressed using Mülliken’s notation:

$$(\mu \nu |\tau \eta )=\int \int \mu (r)\nu (r)\frac{1}{\left|r-r^{\prime }\right|}\tau (r^{\prime })\eta (r^{\prime })drdr^{\prime }$$

#### Excitation Energies

In this work, we propose a Lagrangian for spin-flip excitation energies in the Tamm-Dancoff approximation (TDA),^24^ formulated within the Sternheimer approach, with the aim to derive analytical nuclear coordinate derivatives.^19^ The Lagrangian is defined as an expression for the excitation energies plus two restrictions. These restrictions are the orthonormality of the excitation vectors, **X**, and their orthogonality to the unoccupied β molecular orbitals:

$$G^{\alpha \to \beta }[X,C,\omega ,{\overset{-}{W}}^{X}]=\sum_{i}^{occ}\sum_{\mu }X_{\mu i\alpha }\sum_{j}^{\mathrm{occ}}\sum_{\nu }[F_{\mu \nu \beta }\delta_{ij}-F_{ij\alpha }S_{\mu \nu }]X_{\nu j\alpha }+\sum_{i}^{\mathrm{occ}}\sum_{\mu ,\nu }X_{\mu i\alpha }C_{\nu i\alpha }K_{\mu \nu }^{\mathrm{SF}}[D^{X\alpha \to \beta }]-\omega \left(\sum_{i}^{\mathrm{occ}}\sum_{\mu ,\nu }X_{\mu i\alpha }S_{\mu \nu }X_{\nu i\alpha }-1\right)-\sum_{i,j}^{\mathrm{occ}}{\overset{-}{W}}_{j\beta i\alpha }^{X}\sum_{\mu ,\nu }C_{\mu j\beta }S_{\mu \nu }X_{\nu i\alpha }$$ 1

The proposed Lagrangian depends on the Lagrange multipliers, **W̅** ^*X*^, that are to be determined; excitation vectors, **X**, and excitation energies, ω; and the MO coefficients, **C**. The first term on the right-hand side of eq 1, involving the Kohn–Sham matrices, ***F***, Kronecker delta, δ_*ij*_, and the AO overlap matrix **S**, corresponds to the molecular orbital energy difference in the usual formulation of SF-TDDFT. The Kohn–Sham matrix elements are defined in terms of one-electron integrals, **h**, the ground state density matrix, **D**_σ_, and the exchange-correlation potential, *v*_xc_^σ^:

$$F_{\mu \nu \sigma }=h_{\mu \nu }+\sum_{\sigma^{\prime }}^{\alpha ,\beta }\sum_{\tau ,\eta }((\mu \nu |\tau \eta )-a_{\mathrm{EX}}\delta_{\sigma \sigma \prime }(\mu \tau |\nu \eta ))D_{\tau \eta \sigma \prime }+(\mu |v_{\mathrm{xc}}^{\sigma }|\nu )$$ 2

Here, α_EX_ is a global scaling parameter for the exact exchange. The second term in eq 1 includes the spin-flip kernel matrix **K** ^SF^[**D** ^*X*α→β^], which gathers the noncollinear exchange-correlation functional *f*_xc_^SF^ and the Fock exchange contributions:

$$K_{\mu \nu }^{\mathrm{SF}}[D^{X\alpha \to \beta }]=\sum_{\tau ,\eta }((\mu \nu |f_{\mathrm{xc}}^{\mathrm{SF}}|\tau \eta )-a_{\mathrm{EX}}(\mu \tau |\nu \eta ))D_{\tau \eta }^{X\alpha \to \beta }$$ 3

With the transition density, *D* ^*X*α→β^, defined as

$$D_{\tau \eta }^{X\alpha \to \beta }=\sum_{i}^{\mathrm{occ}}X_{\tau i\alpha }C_{\eta i\alpha }$$ 4

The noncollinear exchange-correlation kernel *f*_xc_^SF^ is defined in terms of the exchange-correlation potential *v*_xc_^σ^.^6^ The noncollinear exchange-correlation kernel contains in the denominator the difference between α and β densities. This denominator can be a source of numerical instabilities and it is known that *f*_xc_^SF^ diverges when employing GGA functionals.^7,8,10^ In this work we propose a modification of the denominator to alleviate the problem:

$$f_{\mathrm{xc}}^{\mathrm{SF}}=\frac{v_{\mathrm{xc}}^{\alpha }(r)-v_{\mathrm{xc}}^{\beta }(r)}{\left|\rho^{\alpha }(r)-\rho^{\beta }(r)\right|}\approx \frac{v_{\mathrm{xc}}^{\alpha }(r)-v_{\mathrm{xc}}^{\beta }(r)}{\max(|\rho^{\alpha }(r)-\rho^{\beta }(r)|,T)}$$ 5

The noncollinear exchange-correlation kernel is implemented using a threshold *T* that is chosen to balance between accuracy and numerical instabilities.

The third term in eq 1 ensures the orthonormality of the excitation vectors **X**, while the fourth term guarantees their orthogonality to the occupied β MO space.

$$\frac{\partial G^{\alpha \to \beta }}{\partial {\mathrm{W̅}}_{j\beta i\alpha }^{X}}=0\to \sum_{\mu ,\nu }C_{\mu j\beta }S_{\mu \nu }X_{\nu i\alpha }=0$$ 6

The Lagrange multipliers **W̅** ^*X*^ are determined from the projection onto the occupied β MO space of the stationary condition of the Lagrangian with respect to variations in **X**:

$$\sum_{\mu }C_{\mu j\beta }\frac{\partial G^{\alpha \to \beta }}{\partial X_{\mu i\alpha }}=0\to {\overset{-}{W}}_{j\beta i\alpha }^{X}=2\sum_{\mu ,\nu }C_{\mu j\beta }K_{\mu \nu }^{\mathrm{SF}}[D^{X\alpha \to \beta }]C_{\nu i\alpha }=2K_{j\beta i\alpha }^{\mathrm{SF}}[D^{X\alpha \to \beta }]$$ 7

Finally, the SF-TDDFT equations in the Sternheimer formulation are derived from the remaining stationary conditions:

$$\frac{\partial G^{\alpha \to \beta }}{\partial X_{\mu i\alpha }}=0\to \sum_{j}^{\mathrm{occ}}\sum_{\nu }[F_{\mu \nu \beta }\delta_{ij}-F_{ij\alpha }S_{\mu \nu }]X_{\nu j\alpha }+\sum_{\nu ,\tau }Q_{\mu \tau \beta }K_{\tau \nu }^{\mathrm{SF}}[D^{X\alpha \to \beta }]C_{\nu i\alpha }=\omega \sum_{\nu }S_{\mu \nu }X_{\nu i\alpha }$$ 8

$$\frac{\partial G^{\alpha \to \beta }}{\partial \omega }=0\to \sum_{i}^{\mathrm{occ}}\sum_{\mu ,\nu }X_{\mu i\alpha }S_{\mu \nu }X_{\nu i\alpha }=1$$ 9

The projection matrix **Q**_β_ is defined as

$$Q_{\mu \tau \beta }=\delta_{\mu \tau }-\sum_{\nu }S_{\mu \nu }\sum_{i}^{\mathrm{occ}}C_{\nu i\beta }C_{\tau i\beta }$$ 10

A notable feature of the Sternheimer formulation of SF-TDDFT is that the eqs 8 and 9 can be transformed into the standard SF-TDDFT expressions by first projecting onto β molecular orbitals and then using the relation:

$$\sum_{a}^{\mathrm{uno}}C_{\mu a\beta }X_{a\beta i\alpha }=X_{\mu i\alpha }$$ 11

For instance, the left-hand side of eq 8 can be rewritten in its standard SF-TDDFT form:

$$\sum_{\mu ,\nu }C_{\mu j\beta }\omega S_{\mu \nu }X_{\nu i\alpha }=\omega \sum_{\mu ,\nu }C_{\mu j\beta }S_{\mu \nu }\sum_{b}^{\mathrm{uno}}C_{\nu b\beta }X_{b\beta i\alpha }=0$$ 12

$$\sum_{\mu ,\nu }C_{\mu a\beta }\omega S_{\mu \nu }X_{\nu i\alpha }=\omega \sum_{\mu ,\nu }C_{\mu a\beta }S_{\mu \nu }\sum_{b}^{\mathrm{uno}}C_{\nu b\beta }X_{b\beta i\alpha }=\omega X_{a\beta i\alpha }$$ 13

This demonstrates the formal equivalence between the Sternheimer and standard formulations.

#### Analytic Derivatives

To eliminate the need for MO derivatives when calculating excitation energy derivatives with respect to an atomic coordinate λ, the Lagrangian in eq 1 must be extended with additional constraints.^19,25,26^ The first constraint enforces the orthonormality of the perturbed MOs and it is enforced by the **W̅** ^*C*^ multipliers and depends on the overlap between σ occupied MOs, *S*_*ij*σ_. The second constraint ensures that the perturbed MOs remain solutions of the noncanonical Kohn–Sham equations, enforced through the Lagrange multiplier **Z̅** ^σ^.

$$L^{\alpha \to \beta }[X,C,\omega ,{\overset{-}{W}}^{X},\overset{-}{Z},{\overset{-}{W}}^{C}]=G^{\alpha \to \beta }[X,C,\omega ,{\overset{-}{W}}^{X}]-\sum_{\sigma }^{\alpha ,\beta }\sum_{i,j}^{\mathrm{occ}}{\mathrm{W̅}}_{ij\sigma }^{C}(S_{ij\sigma }-\delta_{ij})+\sum_{\sigma }^{\alpha ,\beta }\sum_{i}^{\mathrm{occ}}\sum_{\mu ,\nu }{\overset{-}{Z}}_{\mu i\sigma }(F_{\mu \nu \sigma }C_{\nu i\sigma }-S_{\mu \nu }C_{\nu i\sigma }\epsilon_{i\sigma })$$ 14

The Lagrange multipliers **W̅** ^*C*^ are determined by setting the derivative of the Lagrangian in eq 14 with respect to the MO coefficients to zero and projecting the result onto the occupied MO space.

$$\sum_{\mu }\frac{\partial L^{\alpha \to \beta }}{\partial C_{\mu i\sigma }}C_{\mu j\sigma }=0\to {\overset{-}{W}}_{ij\sigma }^{C}=\frac{1}{2}H_{ij\sigma }[P]+\delta_{\sigma \alpha }\sum_{\mu ,\nu }X_{\mu i\alpha }(\omega S_{\mu \nu }-F_{\mu \nu \beta })X_{\nu j\alpha }+g_{ij\sigma }^{\mathrm{SF}}$$ 15

The contributions from the derivative of the Kohn–Sham matrix are captured in **H**[**P**].

$$H_{ij\sigma }[P]=\sum_{\mu ,\nu }C_{\mu i\sigma }H_{\mu \nu \sigma }[P]C_{\mu j\sigma }$$ 16

$$H_{\mu \nu \sigma }[P]=2\sum_{\sigma^{\prime }}^{\alpha ,\beta }\sum_{\tau ,\eta }((\mu \nu |\tau \eta )-a_{\mathrm{EX}}\delta_{\sigma \sigma \prime }(\mu \tau |\nu \eta )+(\mu \nu |f_{\mathrm{xc}}^{\sigma \sigma \prime }|\tau \eta ))P_{\tau \eta \sigma \prime }^{\mathrm{sym}},,P_{\tau \eta \sigma }^{\mathrm{sym}}=\frac{P_{\tau \eta \sigma }+P_{\eta \tau \sigma }}{2}$$ 17

With the relaxed density matrix, **P**, being defined in terms of an unrelaxed difference density matrix, **T**, and a relaxation term, **D** ^*Z*^, defined as

$$P_{\mu \nu \sigma }\equiv T_{\mu \nu \sigma }+D_{\mu \nu \sigma }^{Z}$$ 18

$$T_{\mu \nu \alpha }\equiv -\sum_{i,j}^{\mathrm{occ}}\sum_{\tau ,\eta }C_{\mu i\alpha }X_{\tau i\alpha }S_{\tau \eta }X_{\eta j\alpha }C_{\nu j\alpha }$$ 19

$$T_{\mu \nu \beta }\equiv \sum_{i}^{\mathrm{occ}}X_{\mu i\alpha }X_{\nu i\alpha }$$ 20

$$D_{\mu \nu \sigma }^{Z}\equiv \sum_{i}^{\mathrm{occ}}{\overset{-}{Z}}_{\mu i\sigma }C_{\nu i\sigma }$$ 21

The last term in eq 15 contains the derivative of the noncollinear exchange-correlation functional, it is defined as follows:

$$g_{ij\sigma }^{\mathrm{SF}}=\sum_{\mu ,\nu }\sum_{\tau ,\eta }D_{\mu \nu }^{X}D_{\tau \eta }^{X}(\mu \nu \tau \eta |g_{\mathrm{xc}}^{\sigma \mathrm{SF}}|i^{\sigma }j^{\sigma })$$ 22

And the derivative of the noncollinear exchange-correlation kernel is calculated numerically as

$$g_{\mathrm{xc}}^{\sigma \mathrm{SF}}(r)=,\frac{f^{\mathrm{SF}}\left[\rho^{\sigma }(r)+\frac{\epsilon }{2}D^{X\alpha \to \beta }(r),\rho^{\tau }(r)\right]-f^{\mathrm{SF}}\left[\rho^{\sigma }(r)-\frac{\epsilon }{2}D^{X\alpha \to \beta }(r),\rho^{\tau }(r)\right]}{\epsilon }$$ 23

In eq 23, the response density, *D* ^*X*α→β^(*r*) = ∑_μ,ν_ *D*_μν_^*X*α→β^μ­(*r*)­ν­(*r*), is used to vary the noncollinear kernel at the grid points using the scaling factor ϵ = 10^–3^. The value of ϵ can be modified with the EPS_DELTA_RHO option of the EXCITED_STATE section in the CP2K input.

The multipliers **Z̅** in eq 14 are calculated by solving the Z-vector method equations in the Sternheimer formulation. These equations are obtained by projecting the derivative of *L* with respect to occupied MOs onto the virtual space of the MOs:

$$\sum_{\nu }Q_{\mu \nu \sigma }\frac{\partial L^{\alpha \to \beta }}{\partial C_{\nu i\sigma }}\equiv 0\to \sum_{\nu }(F_{\nu \mu \sigma }-S_{\nu \mu }\epsilon_{i\sigma }){\overset{-}{Z}}_{\nu i\sigma }+\sum_{\nu }Q_{\mu \nu \sigma }H_{\nu i\sigma }[D^{Z}]=-\sum_{\nu }Q_{\mu \nu \sigma }R_{\nu i\sigma }$$ 24

With the response vector elements, *R*_νiσ_, defined as

$$R_{\nu i\sigma }=\sum_{\tau }H_{\nu \tau \sigma }[T]C_{\tau i\sigma }+2\delta_{\sigma \alpha }\sum_{\tau }X_{\tau i\alpha }K_{\tau \nu }^{\mathrm{SF}}[D^{X}]-2\delta_{\sigma \beta }\sum_{\mu }\sum_{j}^{\mathrm{occ}}S_{\nu \mu }X_{\mu j\alpha }K_{i\beta j\alpha }^{\mathrm{SF}}[D^{X\alpha \to \beta }]+2g_{\nu i\sigma }^{\mathrm{SF}}$$ 25

Equation 24 corresponds to the Z-vector method^26^ within the Sternheimer formalism,^17^ adapted for spin-flip excitations. Once the Z-vector is computed, analytical energy derivatives can be calculated as

$$\frac{\partial \omega }{\partial \lambda }=\omega^{\lambda }=\sum_{\sigma }^{\alpha ,\beta }\sum_{\mu ,\nu }(h_{\mu \nu }^{\lambda }+(\mu |v_{xc}^{\sigma }|\nu {)}^{\lambda })P_{\mu \nu \sigma }-\sum_{\mu ,\nu }S_{\mu \nu }^{\lambda }\sum_{\sigma }^{\alpha ,\beta }\Lambda_{\mu \nu \sigma }+\sum_{\mu ,\nu }\sum_{\tau ,\eta }(\mu \nu |\tau \eta {)}^{\lambda }\left[\sum_{\sigma ,\sigma^{\prime }}^{\alpha ,\beta }[P_{\mu \nu \sigma }D_{\tau \eta \sigma \prime }-a_{\mathrm{EX}}\delta_{\sigma \sigma \prime }P_{\mu \tau \sigma }D_{\nu \eta \sigma \prime }]-a_{\mathrm{EX}}D_{\mu \tau }^{X\alpha \to \beta }D_{\nu \eta }^{X\alpha \to \beta }\right]+\sum_{\mu ,\nu }\sum_{\tau ,\eta }(\mu \nu |f_{\mathrm{xc}}^{\mathrm{SF}}|\tau \eta {)}^{\lambda }D_{\mu \nu }^{X\alpha \to \beta }D_{\tau \eta }^{X\alpha \to \beta }$$ 26

Here, **D**_σ_ is the ground state density matrix, and the Pulay term is defined in terms of the following matrix:

$$\Lambda_{\mu \nu \sigma }=\sum_{i,j}^{\mathrm{occ}}X_{\mu i\alpha }(\delta_{\sigma \alpha }F_{ij\alpha }+\delta_{\sigma \beta }\omega \delta_{ij})X_{\nu j\alpha }+\sum_{i}^{\mathrm{occ}}{\mathrm{Z̅}}_{\mu i\sigma }C_{\nu i\sigma }\epsilon_{i\sigma }+\delta_{\sigma \beta }\sum_{i,j}^{\mathrm{occ}}C_{\mu j\beta }K_{j\beta i\alpha }^{\mathrm{SF}}[D^{X\alpha \to \beta }]X_{\nu i\alpha }+\sum_{i,j}^{\mathrm{occ}}C_{\mu i\sigma }{\overset{-}{W}}_{ij\sigma }^{C}C_{\nu j\sigma }$$ 27

### Auxiliary Density Matrix Method

When using functionals that include Fock exchange, the computation of Fock exchange integrals in the operators **H** and **K** ^SF^ can become a significant computational bottleneck. To address this, these integrals can be efficiently approximated using the ADMM.^22,23^ This approach employs a smaller auxiliary basis set for integral calculations while incorporating a correction term based on a local functional, *f*_*x*_, to minimize accuracy loss.^19^ While various projection schemes are available, this work focuses on the nonpurified wave function fitting method, commonly referred to as ADMM2, which will simply be called ADMM here. The use of ADMM in TDDFT has been demonstrated previously^19^; here, we will focus specifically on its implementation for SF-TDDFT. The first term that requires modification is the Fock exchange term in the spin-flip kernel matrix, eq 3,

$$\sum_{\tau ,\eta }(\mu \tau |\nu \eta )D_{\tau \eta }^{X\alpha \to \beta }\approx \sum_{\overset{ˇ}{\gamma },\overset{ˇ}{\delta }}{\overset{ˇ}{U}}_{\overset{ˇ}{\gamma }\mu }{\overset{ˇ}{U}}_{\overset{ˇ}{\delta }\nu }\sum_{\overset{ˇ}{\tau },\overset{ˇ}{\eta }}(\overset{ˇ}{\gamma }\overset{ˇ}{\tau }|\overset{ˇ}{\delta }\overset{ˇ}{\eta }){\overset{ˇ}{D}}_{\overset{ˇ}{\tau }\overset{ˇ}{\eta }}^{X\alpha \to \beta }+\sum_{\tau ,\eta }(\mu \nu |f_{x}[D]|\tau \eta )D_{\tau \eta }^{X\alpha \to \beta }-\sum_{\overset{ˇ}{\mu },\overset{ˇ}{\nu }}{\overset{ˇ}{U}}_{\overset{ˇ}{\mu }\mu }{\overset{ˇ}{U}}_{\overset{ˇ}{\nu }\nu }\sum_{\overset{ˇ}{\tau },\overset{ˇ}{\eta }}(\overset{ˇ}{\mu }\overset{ˇ}{\nu }|f_{x}[\overset{ˇ}{D}]|\overset{ˇ}{\tau }\overset{ˇ}{\eta }){\overset{ˇ}{D}}_{\overset{ˇ}{\tau }\overset{ˇ}{\eta }}^{X\alpha \to \beta }$$ 28

with the projection matrix defined in terms of overlap matrices of atomic orbitals, μ, ν, and the auxiliary functions, μ̌, ν̌ as

$$\overset{ˇ}{U}\equiv {\overset{ˇ}{S}}^{-1}\overset{ˇ}{V}$$ 29

$${\overset{ˇ}{S}}_{\overset{ˇ}{\nu }}\equiv (\overset{ˇ}{\mu }|\overset{ˇ}{\nu })$$ 30

$${\overset{ˇ}{V}}_{\overset{ˇ}{\mu }\nu }\equiv (\overset{ˇ}{\mu }|\nu )$$ 31

The auxiliary matrix, **D** ^*X*α→β^, is obtained by projection of the atomic orbital matrix, **D** ^*X*α→β^,

$${\overset{ˇ}{D}}^{X\alpha \to \beta }=\overset{ˇ}{U}D^{X\alpha \to \beta }{\overset{ˇ}{U}}^{T}$$ 32

and the contribution to the derivatives of Fock exchange integrals in eq 26.

$$\sum_{\mu ,\nu }\sum_{\tau ,\eta }D_{\mu \tau }^{X\alpha \to \beta }(\mu \nu |\tau \eta {)}^{\lambda }D_{\nu \eta }^{X\alpha \to \beta }\approx \sum_{\mu ,\nu }\sum_{\tau ,\eta }\sum_{\overset{ˇ}{\mu },\overset{ˇ}{\nu }}\sum_{\overset{ˇ}{\tau },\overset{ˇ}{\eta }}D_{\mu \tau }^{X\alpha \to \beta }({\overset{ˇ}{U}}_{\overset{ˇ}{\mu }\mu }{\overset{ˇ}{U}}_{\overset{ˇ}{\tau }\tau }{\overset{ˇ}{U}}_{\overset{ˇ}{\nu }\nu }{\overset{ˇ}{U}}_{\overset{ˇ}{\eta }\eta }{)}^{\lambda }((\overset{ˇ}{\mu }\overset{ˇ}{\nu }|\overset{ˇ}{\tau }\overset{ˇ}{\eta })-(\overset{ˇ}{\mu }\overset{ˇ}{\nu }|f_{x}[\overset{ˇ}{D}]|\overset{ˇ}{\tau }\overset{ˇ}{\eta }))D_{\nu \eta }^{X\alpha \to \beta }+\sum_{\overset{ˇ}{\mu },\overset{ˇ}{\nu }}\sum_{\overset{ˇ}{\tau },\overset{ˇ}{\eta }}D_{\overset{ˇ}{\mu }\overset{ˇ}{\tau }}^{X\alpha \to \beta }((\overset{ˇ}{\mu }\overset{ˇ}{\nu }|\overset{ˇ}{\tau }\overset{ˇ}{\eta }{)}^{\lambda }-(\overset{ˇ}{\mu }\overset{ˇ}{\nu }|f_{x}[\overset{ˇ}{D}]|\overset{ˇ}{\tau }\overset{ˇ}{\eta }{)}^{\lambda })D_{\overset{ˇ}{\nu }\overset{ˇ}{\eta }}^{X\alpha \to \beta }+\sum_{\mu ,\nu }\sum_{\tau ,\eta }D_{\mu \tau }^{X\alpha \to \beta }(\mu \nu |f_{x}[D]|\tau \eta {)}^{\lambda }D_{\nu \eta }^{X\alpha \to \beta }$$ 33

With the derivative of the projection matrix being

$${\overset{ˇ}{U}}^{\lambda }={\overset{ˇ}{S}}^{-1}({\overset{ˇ}{V}}^{\lambda }-{\overset{ˇ}{S}}^{\lambda }\overset{ˇ}{U})$$ 34

### Computational Methods

All calculations were carried out using unrestricted Kohn–Sham with a locally modified version of the CP2K package, development version 2024.3.^27^ The validation is divided into four sections, evaluation of the numerical instabilities, vertical excitations, geometry optimizations and ADMM. For all calculations, unless otherwise noted, the following settings were employed: Plane-wave cutoff of 600 Ry and a relative cutoff of 100 Ry. The tolerance for the SCF convergence and for the TDDFT convergence were set to 10^–8^ atomic units. As stated in the Theoretical methods section, all calculations of this work are equivalent to SF-TDDFT with the TDA.

#### Evaluation of the Numerical Instabilities

To assess the numerical stability of the noncollinear kernel, we make use of the zero excitation energy theorem.^16^ According to which the high-spin triplet state calculated in the SCF (*Ms* = 1, ^3^ *T*_1_) is degenerate with the *Ms* = 0 triplet state calculated with SF-TDDFT (*Ms* = 0, ^3^ *T*_0_). Although this degeneracy is not expected to hold for the TDA, numerical tests had shown that the difference in energy between these two states is between 0.01–0.1 eV.^16^ With this in mind, we chose the helium dimer as a testing system. This system was chosen for two reasons. First, its small size allows for the computation of several hundred single-point calculations within a reasonable time. Second, the denominator of the noncollinear kernel, eq 5, approaches zero at many points along the ground-state potential energy surface, as will be shown in the Results and Discussion section. Single point calculations were performed at different bond lengths, every 0.001 Å. At each point, the high-spin triplet state (*Ms* = 1, ^3^ *T*_1_) was calculated in the SCF and the total energy of the excited state (*Ms* = 0, ^3^ *T*_0_) was calculated with SF-TDDFT. Moreover, the analytic energy gradient with respect to nuclear coordinates of the ^3^ *T*_0_ state was calculated at each of these points. The numerical derivative of the excited state energy in between these points was obtained using finite differences following the midpoint rule using the energy calculated at the adjacent points (every 0.001 Å). Consequently, the numerical gradient provides a benchmark for the analytical gradient. The basis set used was DZVP-GTH-PADE with the Goedecker-Teter-Hutter (GTH) pseudopotential GTH-PADE for PADE and PBE functionals, whereas the DZVP-MOL­OPT-PB­E0-GTH-q2/GTH-PBE0-q2 where employed with the PBE0 functional.To speed up these calculations, and considering that accuracy is not relevant for the evaluation of the numerical instability, the plane-wave cutoff was set to 300 Ry with a relative cutoff of 60 Ry. The tolerances of the SCF and TDDFT convergence were set to 10^–7^ atomic units.

#### Vertical Excitations

The molecular geometries and theoretical best estimates (TBE) for the vertical excitations were taken from the QUESTDB database.^28^ From this database, we selected the extrapolations to Full-CI with the largest basis set to excitation energies of singlet and triplet excited states of the so-called “exotic set” of molecules containing diradicals. Excited-state assignments were made by analyzing the transition coefficients in the output. Most of the excited states reported in the QUESTDB used in this work^28^ are dominated by the two singly occupied α MOs and the two lowest unoccupied β MOs. To explain the assignment procedure we temporally use the labels *O*_1α_, *O*_2α_, for the single occupied α MOs and *O*_1β_, *O*_2β_ for the two lowest unoccupied β MOs. Two closed-shell singlet states arise from linear combinations of the excitations *O*_2α_ → *O*_1β_ and *O*_1α_ → *O*_2β_. Of these, the lower-energy state corresponds to the true ground state, while the higher-energy one represents the lowest double excitation from the ground state. Additionally, one open-shell singlet and the ^3^ *T*_0_ triplet state are formed from linear combinations of the excitations *O*_1α_ → *O*_1β_ and *O*_2α_ → *O*_2β_, with the triplet lying nearly degenerate with the ground state (i.e., with excitation energy close to zero). In the subset of the QUESTDB used as reference in this work,^28^ all excitation energies are reported relative to the true ground state. Closed-shell and open-shell excited states are distinguished by their symmetry: closed-shell excitations possess the totally symmetric irreducible representation of the molecular point group.

We tested the PADE^29^ as LDA, PBE,^30^ and PBE0 ^31^ functionals. In every calculation, we employed the GTH pseudopotentials,^29^ along with the corresponding basis set optimized for molecular calculations (MOLOPT). The basis sets tested included DZVP, TZVP, and TZV2P,^32^ each optimized for the specific functional used. For example, in the PBE/DZVP calculations, the DZVP-MOLOPT-PBE-GTH-q4 basis set was employed for the carbon atom. We report the mean signed error (MSE), mean absolute error (MAE), and maximum absolute error (Max) for each functional and basis set combination.

#### Geometry Optimizations

Excited states of a selected set of molecules were optimized and compared to reference structures. The set of molecules comprises of: CH_2_ with FCI/TZ2P reference structures^33^; NH_2_^+^, Si_2_ and PH_2_^+^ with CISD/TZ2P­(f,d) reference structures^34–36^ ; Trimethylmethane (TMM) with collinear SF-TDDFT/50–50/6–311G­(d) reference structures,^37^ the 50–50 functional, combines 50% Hartree–Fock exchange, 8% Slater exchange, 42% Becke exchange, 19% VWN correlation, and 81% LYP correlation.^5^; meta-xylylene with CCSD and EOM-SF-CCSD/6–31G­(d) reference structures^38^; *ortho*-, *meta*- and *para*-benzynes with noncollinear SF-TDD­FT/LDA/cc-pVTZ and SF-CC­SD/cc-pVTZ reference structures.^7^ The optimization of geometries was performed with the conjugated gradient method, employing a tolerance of 10^–3^ a.u. for the maximum and root-mean-square step. The basis set employed was the MOLOPT DZVP.^32^ We tested the functionals PADE as LDA, PBE, PBE0 and PBE with 50% PBE exchange with 50% Fock exchange, referred to as PBE50.

The reference structures are grouped into two groups: High level of theory, comprising of CCSD and CI methods. And SF-TDDFT, including collinear and noncollinear SF-TDDFT. We report the MSE, MAE with respect these two groups and plot the error distribution for bond lengths and angles with respect to the high level of theory group. All the raw data is also reported in tables in the Supporting Information.

#### ADMM

To assess the impact of ADMM on excitation energies, we compared the first 30 excitations of the molecules in Table 1 with and without ADMM using the PBE0 functional. The calculations were performed using the DZVP/dzp, TZVP/tzp, and TZV2P/tz2p basis set/auxiliary basis set combinations. The auxiliary basis sets were taken from the BASIS_ADMM_UZH file in CP2K.^27^ To evaluate the error mitigation of the ADMM correction term (last two terms in eq 28) we compared the calculations using PBE exchange (PBEx) for the correction term with those obtained without (None) the correction term.

**Table 1:** Vertical Excitation Energies [eV] Calculated Using SF-TDDFT versus Theoretical Best Estimates (TBE) from the QUESTDB^28^

		LDA	PBE			PBE0			
molecule	transition	DZVP	DZVP	TZVP	TZV2P	DZVP	TZVP	TZV2P	TBE
F_2_CO	^1^ *A*_1_ → ^1^ *A*_2_	7.62	6.94	6.97	6.92	6.69	6.69	6.67	7.31
	^1^ *A*_1_ → ^3^ *A*_2_	7.22	6.52	6.53	6.49	6.29	6.30	6.27	7.06
CCl_2_	^1^ *A*_1_ → ^1^ *B*_1_	2.36	2.21	2.28	2.25	2.40	2.41	2.39	2.59
	^1^ *A*_1_ → ^1^ *A*_2_	4.13	4.07	4.02	4.04	4.69	4.67	4.71	4.40
	^1^ *A*_1_ → ^3^ *B*_1_	1.18	0.72	0.82	0.81	0.75	0.76	0.76	1.22
	^1^ *A*_1_ → ^3^ *A*_2_	4.13	4.07	4.02	4.04	4.69	4.67	4.71	4.31
CF_2_	^1^ *A*_1_ → ^1^ *B*_1_	4.32	4.16	4.28	4.26	4.57	4.59	4.61	5.09
	^1^ *A*_1_ → ^3^ *B*_1_	2.62	1.89	2.05	2.05	2.05	2.08	2.11	2.77
FHCO	^1^ *A′*→ ^1^ *A*″	6.31	5.71	5.70	5.65	5.43	5.42	5.38	5.96
	^1^ *A′*→ ^3^ *A*″	5.80	5.16	5.14	5.10	4.93	4.92	4.88	5.73
HCCl	^3^ *A*″ → ^1^ *A*″	1.15	1.34	1.36	1.33	1.30	1.30	1.26	1.98
HCF	^3^ *A*″ → ^1^ *A*″	2.20	2.96	2.95	2.94	2.84	2.85	2.80	2.49
HPO	^1^ *A′*→ ^1^ *A*″	2.39	2.33	2.32	2.29	2.29	2.31	2.28	2.47
HPS	^1^ *A′*→ ^1^ *A*″	1.63	1.55	1.55	1.50	1.42	1.43	1.39	1.59
HSiF	^1^ *A′*→ ^1^ *A*″	2.76	2.79	2.84	2.80	2.98	3.02	3.01	3.05
SiCl_2_	^1^ *A*_1_ → ^1^ *B*_1_	3.53	3.53	3.59	3.57	3.80	3.83	3.82	3.91
	^1^ *A*_1_ → ^3^ *B*_1_	2.54	2.18	2.25	2.24	2.23	2.28	2.26	2.48
H_2_CSi	^1^ *A*_1_ → ^1^ *A*_2_	2.71	2.29	2.24	2.22	2.03	1.93	1.91	2.11
	^1^ *A*_1_ → ^1^ *B*_2_	4.11	3.89	3.83	3.78	3.97	3.88	3.80	3.78
MSE		–0.08	–0.31	–0.29	–0.32	–0.26	–0.26	–0.28	
MAE		0.28	0.40	0.36	0.38	0.39	0.38	0.39	

For equilibrium geometry accuracy assessment, we tested ADMM with DZP and TZP auxiliary basis sets. The resulting error distributions for both vertical excitations and equilibrium geometries are presented graphically, while quantitative metrics (MSE and MAE) are tabulated.

## Results and Discussion

### Numerical Stability

From the results obtained with the LDA functional (Figure 1), the following observations can be made: (1) The total energy of the excited state is degenerate with the SCF state, as expected. (2) Both the total energy of the excited state and its analytical derivative exhibit smooth behavior. However, when the PBE functional is employed (inset a in Figure 2): (1) The total energy of the excited state diverges from the SCF reference at certain points. (2) At these bond lengths, both the analytical and numerical derivatives display singularities, indicating numerical instabilities.

**Figure 1:**
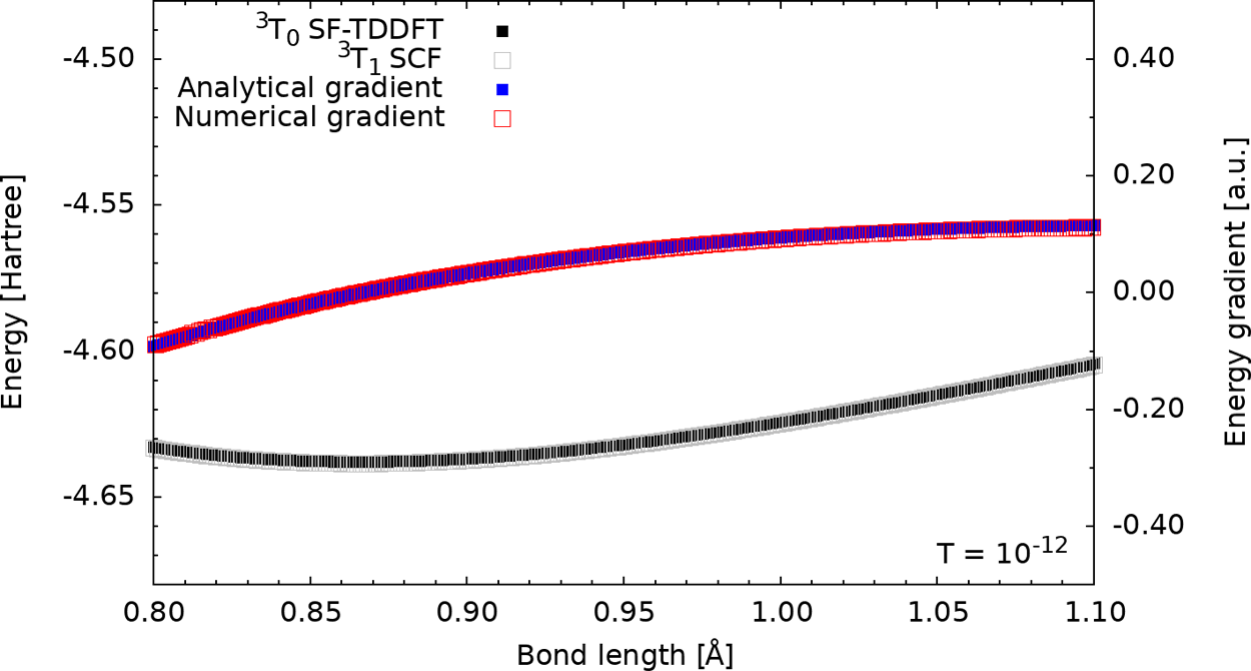
Potential energy surface of the ^3^ *T*_0_ excited state of He_2_ using the PADE functional. Numerical (red) and analytic (blue) gradients are shown.

**Figure 2:**
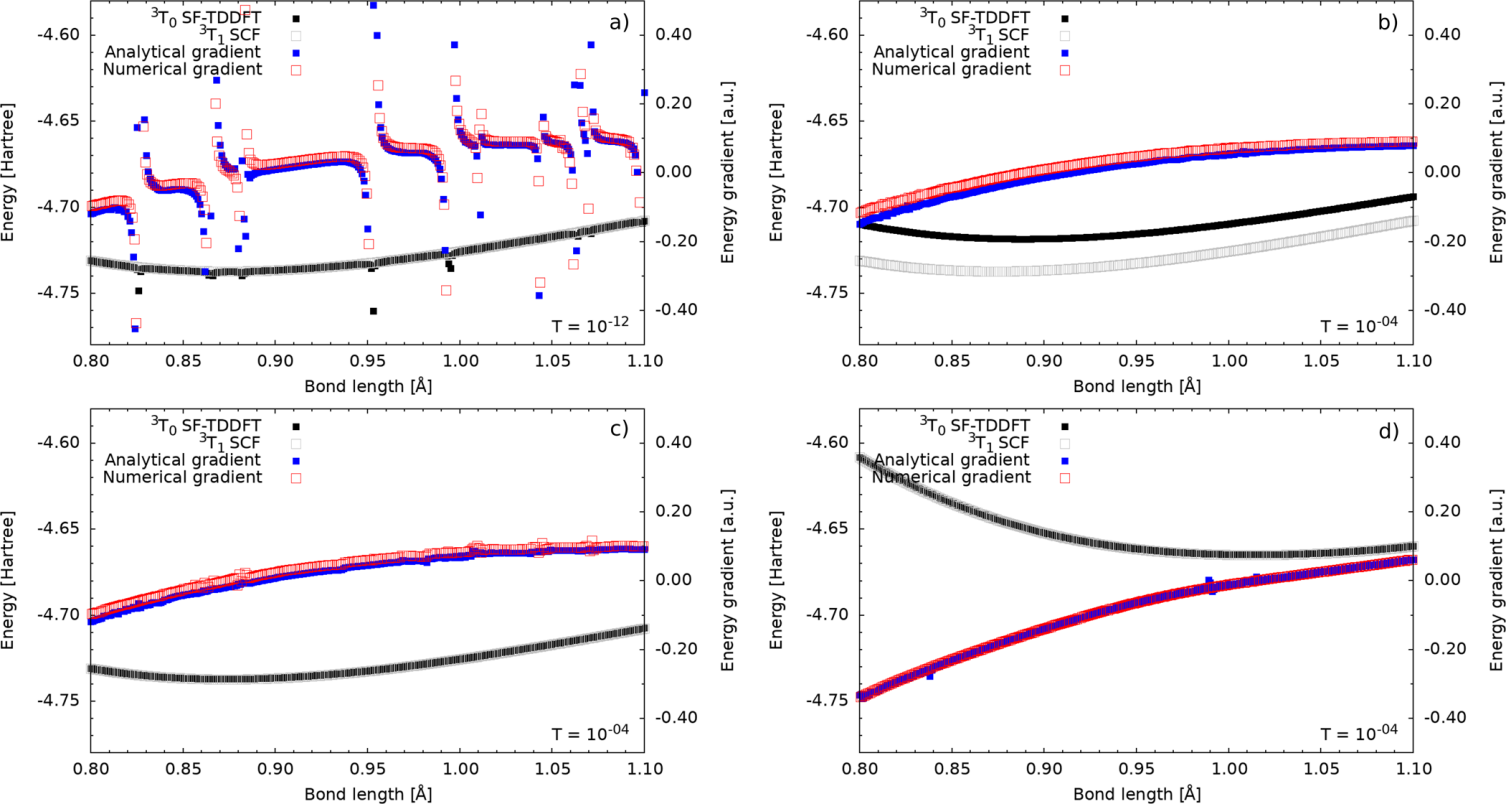
Potential energy surface of the ^3^ *T*_0_ excited state of He_2_ computed using the PBE (insets a and c), PBE with ALDA0 (inset b), and PBE0 (inset d) functionals. Tolerance used for *f*_xc_^SF^ is shown in each inset. Numerical (red) and analytic (blue) gradients are shown for comparison.

To reduce the instabilities observed for PBE, see Figure 2 inset a, we tested different values for the threshold *T* in eq 5. A suitable balance between accuracy and numerical stability was achieved with *T* = 10^–4^. Since this value is relatively large we could not use other stabilization techniques without compromising the accuracy. The other techniques we considered are a constant upshift of the denominator |ρ^α^ – ρ^β^| ≈ |ρ^α^ – ρ^β^| + *T* and screening out grid points where |ρ^α^ – ρ^β^| < *T*. A straightforward approach to mitigate the instability of the GGA noncollinear kernel is to neglect all contributions from the gradient of the density, known as the ALDA0 approximation.^10^ The results of this approach using *T* = 10^–4^, shown in Figure 2 inset b, demonstrate improved numerical stability. However, we observed that the degeneracy with the SCF state is lost regardless of the value of *T*. In Figure 2 inset b we show an energy difference <0.25 eV which is consistent with the energy difference reported in the original ALDA0 publication for the Ethylene molecule.^10^ Due to this lost of degeneracy we consider this solution unsatisfactory. The results for PBE using *T* = 10^–4^ threshold are presented in Figure 2 inset c. Here, the total energy of the excited state is both smooth and degenerate with the SCF energy, as expected. However, the analytical gradient remains slightly unstable. We also tested ϵ = 10^–2^ and ϵ = 10^–4^ in the evaluation of the noncollinear kernel derivative, eq 23, and saw no significant difference.

Additionally, we tested the PBE0 functional (Figure 2 inset d), anticipating improved results due to the inclusion of Fock exchange. Indeed, both the total energy and the analytical gradient exhibited stable and accurate behavior. Using the threshold value *T* = 10^–4^, we achieved a stable and accurate total energy, as shown in Figure 2 inset d. Nevertheless, the gradient calculations, while improved, still exhibit minor inaccuracies and instability.

### Vertical Excitations

The computed vertical excitation energies obtained with different levels of theory, along with the TBE from the reference database,^28^ are presented in Table 1. The overall accuracy of the current implementation is comparable to that of spin-conserving TDDFT for valence excitations of molecules with closed-shell ground states, which typically exhibits errors in the range of 0.3–0.5 eV.^2,3^ This suggests that the use of pseudopotentials and the GPW method does not significantly compromise the accuracy of the computed excitation energies. Notably, the smallest errors are observed in the LDA calculations using the PADE functional. However, the differences between the MSE and MAE values reveal that the errors are more systematic for the PBE and PBE0 functionals. Specifically, these GGA functionals tend to systematically underestimate the excitation energies, which is a known behavior.^2^ Additionally, we observed that the SF-TDDFT excitation energies are relatively insensitive to the size of the basis set. For instance, the MSE for PBE using the DZVP basis set is nearly identical to that obtained with the larger TZV2P basis set.

The PADE functional provides the smallest errors, the systematic underestimation observed with PBE and PBE0 suggests room for improvement in the treatment of exchange-correlation effects. The relative insensitivity to basis set size further underscores the robustness of the SF-TDDFT approach. In summary, the current implementation demonstrates good agreement with reference values, particularly for LDA, while also revealing systematic trends in the performance of PBE and PBE0 functionals.

### Excited State Energy Gradients

All the optimized structure parameters calculated in this work (CH_2_, NH_2_^+^, Si_2_, PH_2_^+^, TMM, *meta*-xylylene, *ortho*-, *meta*- and *para*-benzynes) are summarized in tables in the Supporting Information. The distribution of deviations from high level of theory optimized structure parameters is plotted in Figures 3 and 4. We also report the MSE and MAE with respect to the high level of theory methods and collinear/noncollinear SF-TDDFT references in Table 2.

**Figure 3:**
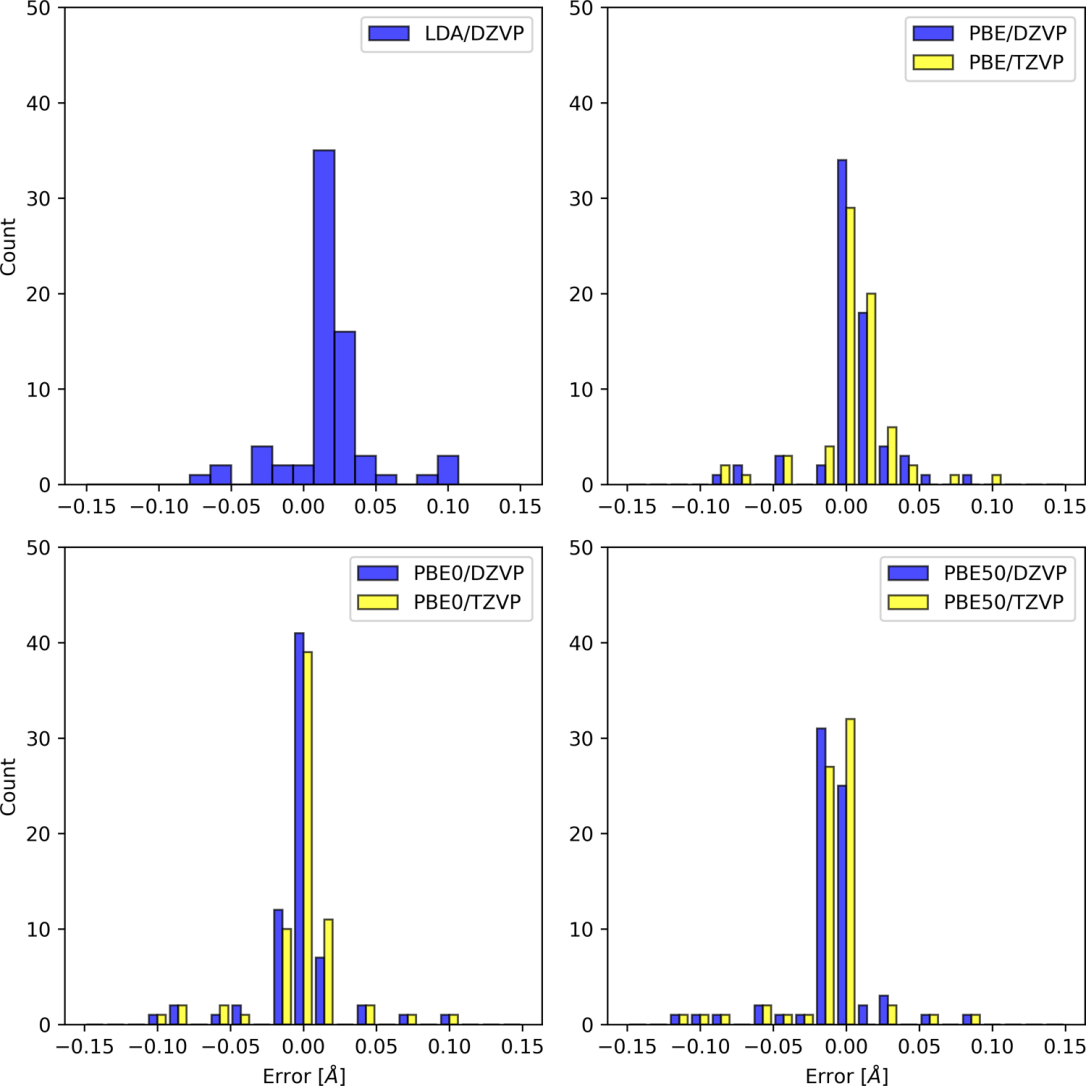
Error distribution for calculated bond lengths with respect to high level of theory methods.

**Figure 4:**
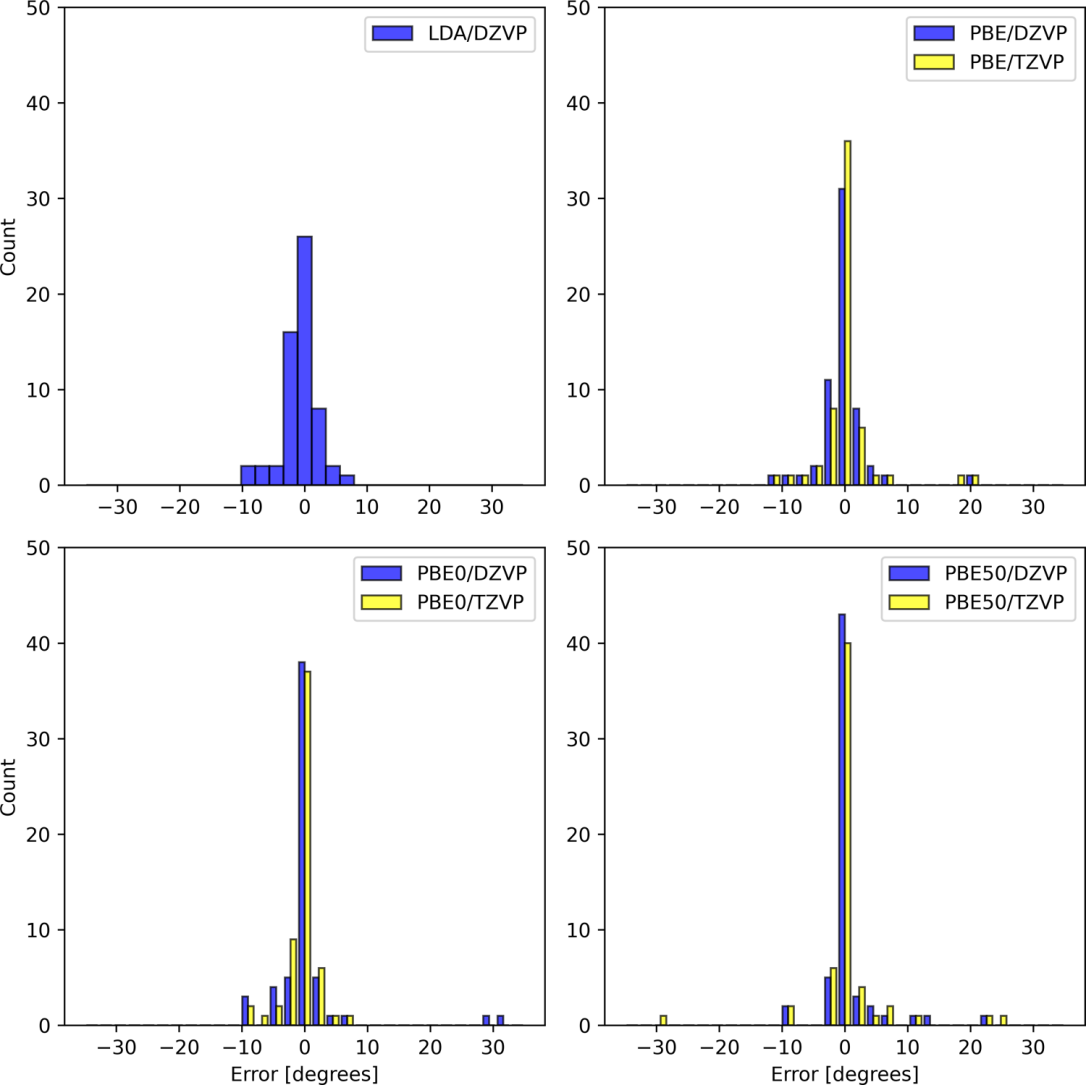
Error distribution for calculated angles using DZVP basis set with respect to high level of theory methods.

**Table 2:** MSE and MAE, in pm, with Respect to Indicated References Using DZVP^a^

	bonds [pm]				angles [°]			
	LDA	PBE	PBE0	PBE50	LDA	PBE	PBE0	PBE50
high-level reference								
MSE	1.7	1.0(0.8)	–0.4(−0.2)	–0.9(−0.9)	–0.8	–0.2(0.2)	0.4(−0.5)	0.7(0.4)
MAE	2.7	2.1(1.9)	1.5(1.5)	1.7(1.6)	2.0	1.9(2.1)	2.5(1.6)	2.0(2.7)
collinear SF-TDDFT/50–50 reference								
MSE	1.9	1.1(1.1)	0.4(0.4)	–0.1(−0.1)	–0.2	0.0(0.0)	0.0(0.0)	0.2(−0.2)
MAE	2.2	1.4(1.9)	1.3(1.3)	1.1(1.0)	0.6	0.4(0.4)	0.4(0.4)	1.1(2.0)
noncollinear SF-TDDFT/LDA reference								
MSE	1.3	0.5(0.6)	–0.3(−0.1)	–0.7(−0.7)	–0.1	0.3(0.1)	0.3(0.0)	0.6(−0.3)
MAE	1.3	0.8(1.7)	0.8(1.2)	1.2(1.1)	0.3	0.8(1.4)	1.3(1.4)	2.7(4.2)

a TZVP results are shown in parentheses.

It is well-established that collinear SF-TDDFT provides accurate equilibrium structures.^5,7^ In our study, the bond lengths obtained were within 2 pm of those reported for collinear SF-TDDFT with the 50–50 functional.^5^ For noncollinear SF-TDDFT, reference equilibrium geometries are currently limited to those obtained with LDA, as numerical instabilities in the noncollinear kernel prevented the use of GGA functionals.^8–10^ Consequently, our comparisons are restricted to noncollinear SF-TDDFT/LDA results. We observed deviations within 1 pm, from the LDA reference, which can be attributed, among others, to differences in basis sets and the use of a pseudopotential in our calculations. These minor discrepancies are consistent with expectations given the methodological variations.

Overal, our results are consistent with the highest accurate reference data (see comparison with High level reference: MAE < 3 pm in Table 2). The performance of PBE0 was remarkably good compared to the High level reference (MSE – 0.4 pm and MAE 1.5 pm Table 2). This result is particularly surprising given the relatively large threshold *T* employed in the numerical integration of the spin-flip kernel.

### ADMM

We evaluated the ADMM for excitation energies and for optimized geometries separately. The error distributions of the excitation energies calculated using the ADMM compared with those calculated without the ADMM are illustrated in Figure 5, while the MSE and MAE are summarized in Table 3.

**Table 3:** Mean Signed Error and Mean Absolute Error [eV] of ADMM Calculations with (PBEx) and without Correction Term (None) Relative to Calculations without the ADMM

	SF-TDDFT^a^ [eV]						TDDFT^b^ [eV]		
	PBEx			none			PBEx		
	DZVP	TZVP	TZV2P	DZVP	TZVP	TZV2P	DZVP	TZVP	TZV2P
MSE	–0.014	0.019	0.019	–0.010	0.020	0.022	–0.064	–0.012	–0.021
MAE	0.052	0.035	0.035	0.113	0.059	0.061	0.094	0.036	0.033

a This work.

b Ref 19.

**Figure 5:**
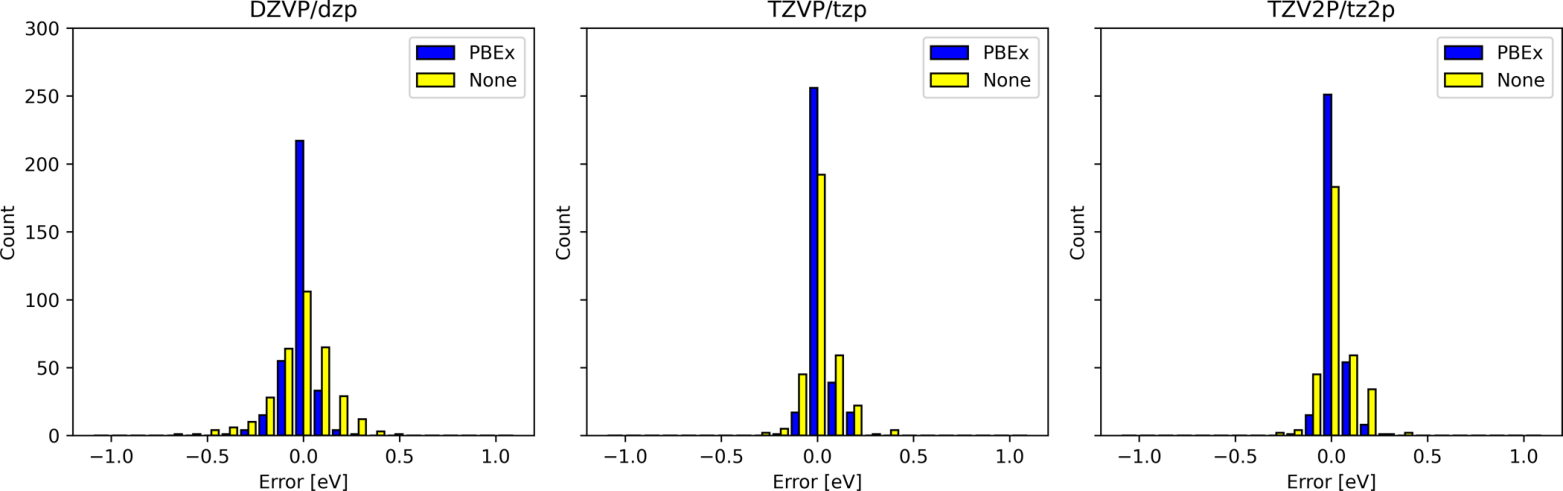
Errors in PBE0 vertical excitation energies relative to calculations without the ADMM. The main basis set/auxiliary basis set is indicated at the top of the figure.

From Table 3 we see that the results improve with increasing basis set size, with the TZVP basis set approaching the basis set limit, as evidenced by the small change relative to TZV2P. Including the correction term reduces, roughly by half, the change in the excitation energy introduced by the ADMM. Overall, the same trends in the performance of the ADMM for excitation energies are observed for SF-TDDFT compared to those of TDDFT.^19^ Given the small difference between TZVP/tzp and TZV2P/tz2p we decided to proceed the validation of optimized geometries with only DZVP/dzp and TZVP/tzp.

The error distributions of calculated bond lengths and angles using the ADMM relative to calculations without the ADMM are shown in Figures 6 and 7, while the MSE and MAE are summarized in Table 4. Interestingly, the inclusion of the correction term does not significantly affect the accuracy of the calculated structures. The MAE for angles when using the ADMM for SF-TDDFT excited state geometry optimizations is larger than those obtained for TDDFT, Table 4. This is due to a few optimized structures having large differences that are of up to 40 degrees, see Figure 7, with respect to optimizations without the ADMM. The reason for these outliers likely is the quality of the noncollinear kernel that is affected with the large threshold in eq 5.

**Table 4:** Mean Signed Error and Mean Absolute Error of ADMM Optimized Geometries with (PBEx) and without Correction Term (None) with Respect to Non-ADMM Calculations

	SF-TDDFT^a^				TDDFT^b^	
	PBEx		none		PBEx	
	DZVP	TZVP	DZVP	TZVP	DZVP	TZVP
bonds [pm]						
MSE	0.8	0.3	1.1	0.3	1.3	0.2
MAE	1.2	1.1	1.4	1.0	1.3	0.2
angles [°]						
MSE	–0.2	0.7	–1.2	0.3	–0.7	–0.1
MAE	2.5	2.2	2.4	1.6	0.9	0.2

a This work.

b Ref 19.

**Figure 6:**
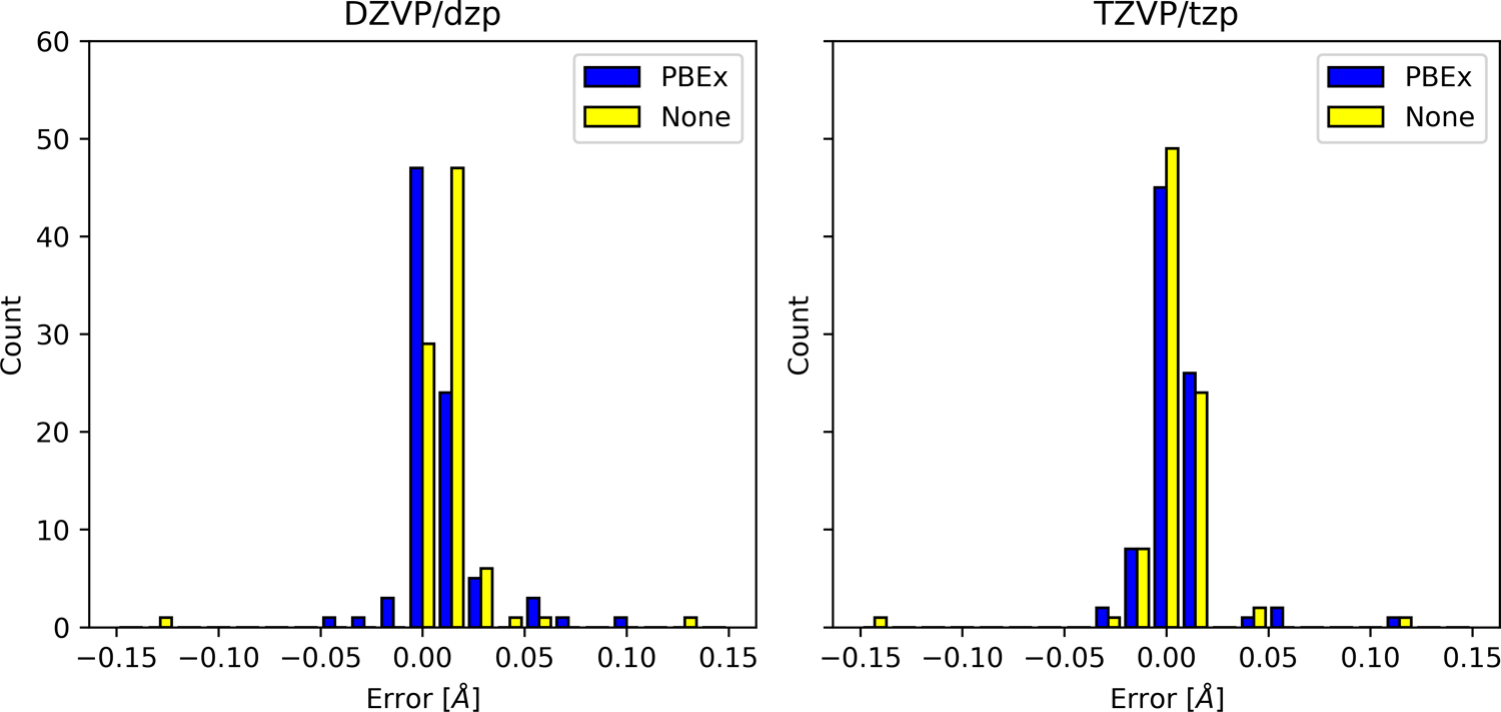
Errors in PBE0 bonds of equilibrium geometries relative to calculations without the ADMM. The main basis set/auxiliary basis set is indicated at the top of the figure.

**Figure 7:**
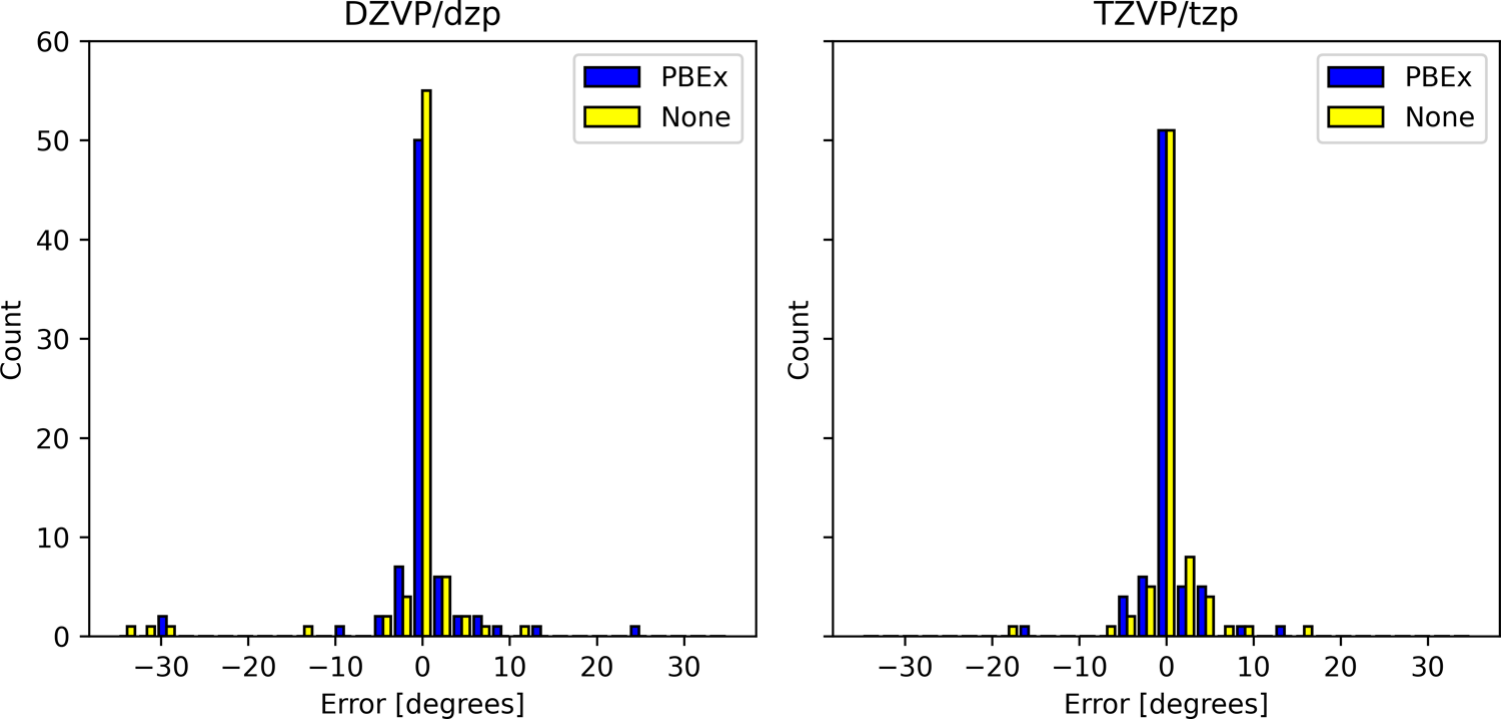
Errors in PBE0 angles of equilibrium geometries relative to calculations without the ADMM. The main basis set/auxiliary basis set is indicated at the top of the figure.

In this work, energy and geometry optimization calculations using ADMM were generally slower than those performed without it. This behavior is expected due to the additional operations introduced by the ADMM formalism. However, the efficiency gains of ADMM are anticipated to become significant for systems with approximately 100 atoms or more.^19^

## Conclusions

In this work, we presented the first implementation of SF-TDDFT within the Sternheimer formulation including its extension to the GPW, needing only a few unoccupied molecular orbitals. The implementation leverages the GPW method and is integrated into the CP2K package featuring GPW. Key features of our approach include the use of a noncollinear exchange-correlation kernel, stabilization techniques for GGA functionals, and the extension to the ADMM to reduce computational costs.

Our benchmark calculations for vertical excitation energies demonstrated that the implementation yields results consistent with high-level reference data from the QUESTDB database. The LDA functional (PADE) exhibited the smallest errors, while PBE and PBE0 showed systematic underestimation of excitation energies, with MAE of approximately 0.4 eV.

For geometry optimizations, the PBE0 functional in particular, delivered outstanding accuracy, with mean deviations of bond lengths as small as 0.004 Å. The ADMM approximation introduced only minor errors, with bond length deviations of around 0.01 Å, making it a viable option for large-scale applications. Overall, the noncollinear kernel produced equilibrium structures in excellent agreement with high-level reference data, including CCSD, CISD, and FCI results.

Despite the numerical challenges associated with the noncollinear kernel for GGA functionals, our stabilization strategy, employing a threshold *T* = 10^–4^, ensured smooth potential energy surfaces and reliable gradients. While minor instabilities in the analytical gradients persist, the overall performance of the implementation is promising, especially for hybrid functionals like PBE0. The expected degeneracy between the ^3^ *T*_1_ and ^3^ *T*_0_ states was preserved to within 10^–2^ eV for the PADE, PBE, and PBE0 functionals. This energy splitting is of the same order of magnitude as the shift introduced by the TDA itself.^16^

In summary, this work advances the practical application of SF-TDDFT by providing a computationally efficient and accurate framework for studying excited-state properties and geometries of systems with multireference character. Future developments could focus on further stabilizing the noncollinear kernel for GGA functionals and extending the implementation to more complex systems, including periodic materials and solvated environments.

## Supplementary Material



## Notes

Determination of all the Lagrange multipliers and the optimized geometrical parameters of all molecules (PDF)
