# Supplementary material for: Spin-Flip TDDFT within the Sternheimer Formulation: A Gaussian and Plane Wave Implementation
Source: J Phys Chem A. 2025 Oct 14;129(42):9798–809. doi: 10.1021/acs.jpca.5c05234 (PMC12557386; doi:10.1021/acs.jpca.5c05234)
Supplement: Supplementary file 1 [file jp5c05234_si_001.pdf]

Spin-flip TDDFT within the Sternheimer formulation: a Gaussian and plane wave implementation

Luis I. Hernandez-Segura* and Sandra Luber

University of Zürich, Department of Chemistry, Zürich, 8057, Switzerland.

E-mail: luisignacio.hernandezsegura@uzh.ch

Content

- Determination of the Lagrange multipliers
- Optimized parameters

Determination of the Lagrange multipliers

Determination of the Lagrange multipliers $\bar{\boldsymbol{W}}^X$

We start by expanding the derivative:

$$\begin{aligned}\sum_{\mu} C_{\mu j\beta}\frac{\partial G^{\alpha\to\beta}}{\partial X_{\mu i\alpha}} &= \sum_{\mu} C_{\mu j\beta}\frac{\partial}{\partial X_{\mu i\alpha}}\sum_{i}^{occ}\sum_{\mu} X_{\mu i\alpha}\sum_{k}^{occ}\sum_{\nu}\left[F_{\mu\nu\beta}\delta_{ik} - F_{ik\alpha}S_{\mu\nu}\right]X_{\nu k\alpha} \\ &- \omega\sum_{\mu} C_{\mu j\beta}\frac{\partial}{\partial X_{\mu i\alpha}}\left(\sum_{i}^{occ}\sum_{\mu,\nu} X_{\mu i\alpha}S_{\mu\nu}X_{\nu i\alpha} - 1\right) \\ &+ \sum_{\mu} C_{\mu j\beta}\frac{\partial}{\partial X_{\mu i\alpha}}\sum_{i}^{occ}\sum_{\mu,\nu} X_{\mu i\alpha}C_{\nu i\alpha}K^{SF}_{\mu\nu}[\boldsymbol{D}^{X\alpha\to\beta}] \\ &- \sum_{\mu} C_{\mu j\beta}\frac{\partial}{\partial X_{\mu i\alpha}}\sum_{i,k}^{occ}\bar{W}^X_{k\beta i\alpha}\sum_{\mu,\nu} C_{\nu k\beta}S_{\nu\mu}X_{\mu i\alpha} \\ &= 2\sum_{\mu} C_{\mu j\beta}\sum_{k}^{occ}\sum_{\nu}\left[F_{\mu\nu\beta}\delta_{ik} - F_{ik\alpha}S_{\mu\nu}\right]X_{\nu k\alpha} - 2\omega\sum_{\mu,\nu} C_{\mu j\beta}S_{\mu\nu}X_{\nu i\alpha} \\ &+ \sum_{\mu,\nu} C_{\mu j\beta}C_{\nu i\alpha}K^{SF}_{\mu\nu}[\boldsymbol{D}^{X\alpha\to\beta}] + \sum_{\mu}\sum_{\nu,\eta} C_{\mu j\beta}X_{\nu i\alpha}C_{\eta i\alpha}\frac{\partial K^{SF}_{\nu\eta}[\boldsymbol{D}^{X\alpha\to\beta}]}{\partial X_{\mu i\alpha}} \\ &- \sum_{k}^{occ}\bar{W}^X_{k\beta i\alpha}\sum_{\mu,\nu} C_{\nu k\beta}S_{\nu\mu}C_{\mu j\beta} = 0 \end{aligned} \tag{S1}$$

We now assume $\boldsymbol{X}$ to be orthogonal to the unoccupied β MO space. Thus, the first two terms vanish. We now realize that the third and fourth terms are the same by expanding the derivative of the kernel matrix, $\boldsymbol{K}^{SF}$:

$$\begin{aligned}\sum_{\mu}\sum_{\nu,\eta} C_{\mu j\beta}X_{\nu i\alpha}C_{\eta i\alpha}\frac{\partial K^{SF}_{\nu\eta}[\boldsymbol{D}^{X\alpha\to\beta}]}{\partial X_{\mu i\alpha}} &= \sum_{\mu,\nu}\sum_{\eta}\sum_{\lambda} X_{\nu i\alpha}C_{\eta i\alpha}\Big((\nu\eta|f^{SF}_{xc}|\mu\lambda) - a_{EX}(\nu\mu|\eta\lambda)\Big)C_{\mu j\beta}C_{\lambda i\alpha} \\ &= \sum_{\mu,\lambda} C_{\mu j\beta}C_{\lambda i\alpha}K^{SF}_{\mu\lambda}[\boldsymbol{D}^{X\alpha\to\beta}] = K^{SF}_{j\beta i\alpha}[\boldsymbol{D}^{X\alpha\to\beta}]\end{aligned} \tag{S2}$$

After solving eq. (S1) for $\boldsymbol{W}^X$ we find the final result:

$$\bar{W}^X_{j\beta i\alpha} = 2K^{SF}_{j\beta i\alpha}[\boldsymbol{D}^{X\alpha\rightarrow\beta}] \tag{S3}$$

Determination of the $\boldsymbol{X}$ multipliers

Expanding the derivative yields:

$$\frac{\partial G^{\alpha\rightarrow\beta}}{\partial X_{\mu i\alpha}} = 2\sum_j^{occ}\sum_\nu \left[F_{\mu\nu\beta}\delta_{ij} - F_{ij\alpha}S_{\mu\nu}\right]X_{\nu j\alpha} + 2\sum_\nu K^{SF}_{\mu\nu}[\boldsymbol{D}^{X\alpha\rightarrow\beta}]C_{\nu i\alpha} \tag{S4}$$

$$-2\omega\sum_\nu S_{\mu\nu}X_{\nu i\alpha} - \sum_j^{occ}\bar{W}^X_{j\beta i\alpha}\sum_\nu C_{\nu j\beta}S_{\nu\mu} - \sum_{k,j}^{occ}\frac{\partial\bar{W}^X_{j\beta k\alpha}}{\partial X_{\mu i\alpha}}\sum_{\eta,\nu}C_{\nu j\beta}S_{\nu\eta}X_{\eta k\alpha} = 0 \tag{S5}$$

By using the definition of the $\boldsymbol{W}^X$ multiplier, we can see that:

$$\sum_j^{occ}\bar{W}^X_{j\beta i\alpha}\sum_\nu C_{\nu j\beta}S_{\nu\mu} = 2\sum_j^{occ}K^{SF}_{j\beta i\alpha}[\boldsymbol{D}^{X\alpha\rightarrow\beta}]\sum_\eta C_{\eta j\beta}S_{\eta\mu} = 2\sum_{\eta,\tau}S_{\mu\eta}\sum_j^{occ}C_{\eta j\beta}C_{\tau j\beta}\sum_\nu K^{SF}_{\tau\nu}[\boldsymbol{D}^{X\alpha\rightarrow\beta}]C_{\nu i\alpha} \tag{S6}$$

$$\sum_{k,j}^{occ}\frac{\partial\bar{W}^X_{j\beta k\alpha}}{\partial X_{\mu i\alpha}}\sum_{\eta,\nu}C_{\nu j\beta}S_{\nu\eta}X_{\eta k\alpha} = 0 \tag{S7}$$

If we define a projection matrix we can combine the following two terms:

$$\begin{aligned}\sum_\nu K^{SF}_{\mu\nu}[\boldsymbol{D}^{X\alpha\rightarrow\beta}]C_{\nu i\alpha} - \sum_{\eta,\tau}S_{\mu\eta}\sum_j^{occ}C_{\eta j\beta}C_{\tau j\beta}\sum_\nu K^{SF}_{\tau\nu}[\boldsymbol{D}^{X\alpha\rightarrow\beta}]C_{\nu i\alpha}&\\ = \sum_\tau\delta_{\mu\tau}\sum_\nu K^{SF}_{\tau\nu}[\boldsymbol{D}^{X\alpha\rightarrow\beta}]C_{\nu i\alpha} - \sum_\tau\left[\sum_\eta S_{\mu\eta}\sum_j^{occ}C_{\eta j\beta}C_{\tau j\beta}\right]\sum_\nu K^{SF}_{\tau\nu}[\boldsymbol{D}^{X\alpha\rightarrow\beta}]C_{\nu i\alpha}&\\ = \sum_\tau Q_{\mu\tau\beta}\sum_\nu K^{SF}_{\tau\nu}[\boldsymbol{D}^{X\alpha\rightarrow\beta}]C_{\nu i\alpha}&\end{aligned} \tag{S8}$$

With

$$Q_{\mu\tau\beta} = \delta_{\mu\tau} - \sum_\eta S_{\mu\eta}\sum_j^{occ}C_{\eta j\beta}C_{\tau j\beta} \tag{S9}$$

Thus, we can determine $\boldsymbol{X}$ by solving:

$$\sum_{j}^{occ}\sum_{\nu}\left[F_{\mu\nu\beta}\delta_{ij}-F_{ij\alpha}S_{\mu\nu}\right]X_{\nu j\alpha}+\sum_{\nu,\tau}Q_{\mu\tau\beta}K^{SF}_{\tau\nu}[\boldsymbol{D}^{X\alpha\rightarrow\beta}]C_{\nu i\alpha}=\omega\sum_{\nu}S_{\mu\nu}X_{\nu i\alpha} \tag{S10}$$

Determination of the $\bar{\boldsymbol{W}}^C$ multipliers

$$\begin{aligned}\sum_{\mu}\frac{\partial L^{\alpha\rightarrow\beta}}{\partial C_{\mu i\sigma}}C_{\mu j\sigma}&=\sum_{\mu}\frac{\partial G^{\alpha\rightarrow\beta}}{\partial C_{\mu i\sigma}}C_{\mu j\sigma}-\sum_{\mu}\sum_{k}^{occ}\frac{\partial S_{ik\sigma}}{\partial C_{\mu i\sigma}}C_{\mu j\sigma}\bar{W}^C_{ik\sigma}\\&+\sum_{\mu,\nu}C_{\mu j\sigma}\frac{\partial}{\partial C_{\mu i}}\sum_{k}^{occ}\sum_{\tau}\bar{Z}_{\nu k\sigma}\left(F_{\nu\tau\sigma}-S_{\nu\tau}\epsilon_{k\sigma}\right)C_{\tau k\sigma}=0\end{aligned} \tag{S11}$$

The second term in eq. (S11) is just the $\bar{\boldsymbol{W}}^C$ multiplier:

$$\sum_{\mu}\sum_{k}^{occ}\frac{\partial S_{ik\sigma}}{\partial C_{\mu i\sigma}}C_{\mu j\sigma}\bar{W}^C_{ik\sigma}=2\sum_{\mu,\nu}\sum_{k}^{occ}C_{\mu j\sigma}S_{\mu\nu}C_{\nu k\sigma}\bar{W}^C_{ik\sigma}=2\bar{W}^C_{ij\sigma} \tag{S12}$$

The third term in eq. (S11) contains the contribution from the Z-vector.

$$\begin{aligned}\sum_{\mu,\nu}C_{\mu j\sigma}\frac{\partial}{\partial C_{\mu i}}\sum_{k}^{occ}\sum_{\tau}\bar{Z}_{\nu k\sigma}\left(F_{\nu\tau\sigma}-S_{\nu\tau}\epsilon_{k\sigma}\right)C_{\tau k\sigma}&=\sum_{\mu,\nu}C_{\mu j\sigma}\sum_{k}^{occ}\sum_{\tau}\bar{Z}_{\nu k\sigma}\frac{\partial F_{\nu\tau\sigma}}{\partial C_{\mu i}}C_{\tau k\sigma}\\&+\sum_{\mu,\nu}\bar{Z}_{\nu i\sigma}\left(F_{\nu\mu\sigma}-S_{\nu\mu}\epsilon_{i\sigma}\right)C_{\mu j\sigma}=H_{ij}[\boldsymbol{D}^Z]\end{aligned} \tag{S13}$$

The first term on the r.h.s of eq. (S11) will be split into three contributions as shown below.

$$\begin{aligned}\sum_{\mu}\frac{\partial G^{\alpha\rightarrow\beta}}{\partial C_{\mu i\sigma}}C_{\mu j\sigma}&=\sum_{k}^{occ}\sum_{\mu,\nu}X_{\nu k\alpha}\sum_{l}^{occ}\sum_{\eta}\frac{\partial\left[F_{\nu\eta\beta}\delta_{kl}-F_{kl\alpha}S_{\nu\eta}\right]}{\partial C_{\mu i\sigma}}C_{\mu j\sigma}X_{\eta l\alpha}\\&+\sum_{k}^{occ}\sum_{\nu,\eta}\sum_{\mu,\lambda}X_{\nu k\alpha}Q_{\nu\lambda\beta}\frac{\partial K^{SF}_{\lambda\eta}[\boldsymbol{D}^{X\alpha\rightarrow\beta}]C_{\eta k\alpha}}{\partial C_{\mu i\sigma}}C_{\mu j\sigma}\\&-\sum_{k}^{occ}\sum_{\nu,\eta}\sum_{\mu,\lambda}X_{\nu k\alpha}K^{SF}_{\lambda\eta}[\boldsymbol{D}^{X\alpha\rightarrow\beta}]C_{\eta k\alpha}\frac{\partial Q_{\nu\lambda\beta}}{\partial C_{\mu i\sigma}}C_{\mu j\sigma}\\&-2\sum_{k,l}^{occ}\sum_{\nu,\eta}\sum_{\mu}\frac{\partial K^{SF}_{l\beta k\alpha}[\boldsymbol{D}^{X\alpha\rightarrow\beta}]C_{\nu l\beta}}{\partial C_{\mu i\sigma}}C_{\mu j\sigma}S_{\nu\eta}X_{\eta k\alpha}\end{aligned} \tag{S14}$$

The first contribution can be rearranged in the following way

$$\sum_{k}^{occ}\sum_{\mu,\nu} X_{\nu k\alpha} \sum_{l}^{occ}\sum_{\eta} \frac{\partial\left[F_{\nu\eta\beta}\delta_{kl} - F_{kl\alpha}S_{\nu\eta}\right]}{\partial C_{\mu i\sigma}} C_{\mu j\sigma} X_{\eta l\alpha} = \sum_{\mu,\nu}\sum_{\eta} \frac{\partial F_{\nu\eta\beta}}{\partial C_{\mu i\sigma}} C_{\mu j\sigma} \sum_{k}^{occ} X_{\nu k\alpha} X_{\eta k\alpha}$$
$$- \sum_{\mu}\sum_{\lambda,\tau} \frac{\partial F_{\lambda\tau\alpha}}{\partial C_{\mu i\sigma}} C_{\mu j\sigma} \sum_{k,l}^{occ}\sum_{\eta,\nu} C_{\lambda k\alpha} X_{\nu k\alpha} S_{\nu\eta} X_{\eta l\alpha} C_{\tau l\alpha}$$
$$- \sum_{\mu} C_{\mu j\sigma} \sum_{\lambda,\tau} F_{\lambda\tau\alpha} \sum_{k,l}^{occ} \frac{\partial\left[C_{\lambda k\alpha}C_{\tau l\alpha}\right]}{\partial C_{\mu i\sigma}} \sum_{\eta,\nu} X_{\nu k\alpha} S_{\nu\eta} X_{\eta l\alpha} \tag{S15}$$

The first two terms on the r.h.s. of eq. (S15) can be combined into one:

$$\sum_{\mu,\nu}\sum_{\eta} \frac{\partial F_{\nu\eta\beta}}{\partial C_{\mu i\sigma}} C_{\mu j\sigma} \sum_{k}^{occ} X_{\nu k\alpha} X_{\eta k\alpha} - \sum_{\mu}\sum_{\lambda,\tau} \frac{\partial F_{\lambda\tau\alpha}}{\partial C_{\mu i\sigma}} C_{\mu j\sigma} \sum_{k,l}^{occ}\sum_{\eta,\nu} C_{\lambda k\alpha} X_{\nu k\alpha} S_{\nu\eta} X_{\eta l\alpha} C_{\tau l\alpha}$$
$$= \sum_{\sigma'}\sum_{\mu,\nu}\sum_{\eta} \frac{\partial F_{\nu\eta\sigma'}}{\partial C_{\mu i\sigma}} C_{\mu j\sigma} T_{\nu\eta\sigma'}$$
$$= 2\sum_{\sigma'}\sum_{\mu,\nu}\left[(i\sigma j\sigma|\mu\nu) - \frac{\delta_{\sigma\sigma'}a_{EX}}{2}\Big\{(i\sigma\mu|j\sigma\nu) + (i\sigma\nu|j\sigma\mu)\Big\} + (i\sigma j\sigma|f_{xc}^{\sigma'}|\mu\nu)\right] T_{\mu\nu\sigma'}$$
$$= H_{ij\sigma}[\boldsymbol{T}] \tag{S16}$$

And the last term of eq. (S15) is

$$\sum_{\mu} C_{\mu j\sigma} \sum_{\lambda,\tau} F_{\lambda\tau\alpha} \sum_{k,l}^{occ} \frac{\partial\left[C_{\lambda k\alpha}C_{\tau l\alpha}\right]}{\partial C_{\mu i\sigma}} \sum_{\eta,\nu} X_{\nu k\alpha} S_{\nu\eta} X_{\eta l\alpha} = 2\delta_{\alpha\sigma} \sum_{k}^{occ} F_{jk\alpha} \sum_{\eta,\nu} X_{\nu i\alpha} S_{\nu\eta} X_{\eta k\alpha} \tag{S17}$$

The second term in eq. (S14) contains the derivative of the noncollinear exchange-correlation kernel:

$$\sum_{k}^{occ}\sum_{\nu,\eta}\sum_{\mu,\lambda} X_{\nu k\alpha} Q_{\nu\lambda\beta} \frac{\partial K_{\lambda\eta}^{SF}[\boldsymbol{D}^{X\alpha\to\beta}] C_{\eta k\alpha}}{\partial C_{\mu i\sigma}} C_{\mu j\sigma} =$$
$$2\delta_{\alpha\sigma}\sum_{\nu}\sum_{\mu,\lambda} X_{\nu i\alpha} Q_{\nu\lambda\beta} K_{\lambda\mu}^{SF}[\boldsymbol{D}^{X\alpha\to\beta}] C_{\mu j\sigma} + 2\sum_{\mu,\nu}\sum_{\tau,\eta} D_{\mu\nu}^{X\alpha\to\beta} D_{\tau\eta}^{X\alpha\to\beta} (\mu\nu\tau\eta|g_{xc}^{\sigma SF}|i^{\sigma}j^{\sigma}) \tag{S18}$$

The third term in eq. (S14) vanishes due to the orthogonality of the excitation vector to the occupied β space.

$$\sum_{k}^{occ}\sum_{\nu,\eta}\sum_{\mu,\lambda} C_{\eta k\alpha} K^{SF}_{\lambda\eta}[\boldsymbol{D}^{X\alpha\to\beta}] X_{\nu k\alpha} \frac{\partial Q_{\nu\lambda\beta}}{\partial C_{\mu i\sigma}} C_{\mu j\sigma} = -\,\delta_{\beta\sigma}\sum_{k}^{occ}\sum_{\nu,\eta}\sum_{\mu,\lambda} C_{\eta k\alpha} K^{SF}_{\lambda\eta}[\boldsymbol{D}^{X\alpha\to\beta}] X_{\nu k\alpha} S_{\nu\mu} C_{\lambda i\beta} C_{\mu j\sigma} - \delta_{\beta\sigma}\sum_{k}^{occ}\sum_{\nu,\eta}\sum_{\mu,\tau} C_{\eta k\alpha} K^{SF}_{\mu\eta}[\boldsymbol{D}^{X\alpha\to\beta}] X_{\nu k\alpha} S_{\nu\tau} C_{\tau i\beta} C_{\mu j\sigma} = 0 \tag{S19}$$

Similar, the last term in eq. (S14) vanishes:

$$\sum_{k,l}^{occ}\sum_{\nu,\eta}\sum_{\mu} \frac{\partial K^{SF}_{l\beta k\alpha}[\boldsymbol{D}^{X\alpha\to\beta}] C_{\nu l\beta}}{\partial C_{\mu i\sigma}} C_{\mu j\sigma} S_{\nu\eta} X_{\eta k\alpha} = \sum_{k}^{occ} K^{SF}_{i\beta k\alpha}[\boldsymbol{D}^{X\alpha\to\beta}]\delta_{\beta\sigma}\sum_{\eta,\mu} C_{\mu j\sigma} S_{\mu\eta} X_{\eta k\alpha} + \sum_{k,l}^{occ}\sum_{\mu}\frac{\partial K^{SF}_{l\beta k\alpha}[\boldsymbol{D}^{X\alpha\to\beta}]}{\partial C_{\mu i\sigma}} C_{\mu j\sigma}\sum_{\nu,\eta} C_{\nu l\beta} S_{\nu\eta} X_{\eta k\alpha} = 0 \tag{S20}$$

Thus, we find that:

$$\begin{aligned}\bar{W}^{C}_{ij\sigma} &= \frac{1}{2}H_{ij\sigma}[\boldsymbol{D}^{Z}] + g^{SF}_{ij\sigma} + \delta_{\alpha\sigma}\sum_{\nu} X_{\nu i\alpha}\left[-\sum_{k}^{occ} F_{jk\alpha}\sum_{\eta} S_{\nu\eta} X_{\eta k\alpha} + \sum_{\mu,\eta} Q_{\nu\eta\beta} K^{SF}_{\eta\mu}[\boldsymbol{D}^{X\alpha\to\beta}] C_{\mu j\sigma}\right] \\ &= \frac{1}{2}H_{ij\sigma}[\boldsymbol{D}^{Z}] + g^{SF}_{ij\sigma} + \delta_{\alpha\sigma}\sum_{\nu,\eta} X_{\nu i\alpha}\left[\omega S_{\nu\eta} - F_{\nu\eta\beta}\right] X_{\eta j\alpha}\end{aligned} \tag{S21}$$

Determination of the $\bar{\boldsymbol{Z}}$ multipliers

$$\sum_{\nu} Q_{\mu\nu\sigma}\frac{\partial L^{\alpha\to\beta}}{\partial C_{\nu i\sigma}} = \sum_{\nu} Q_{\mu\nu\sigma}\frac{\partial G^{\alpha\to\beta}}{\partial C_{\nu i\sigma}} - \sum_{\sigma'}^{\alpha,\beta}\sum_{k,j}^{occ}\sum_{\nu} Q_{\mu\nu\sigma}\frac{\partial S_{kj\sigma'}}{\partial C_{\nu i\sigma}}\bar{W}^{C}_{kj\sigma'} + \sum_{\eta,\nu} Q_{\mu\nu\sigma}\left(F_{\eta\nu\sigma} - S_{\eta\nu}\epsilon_{i\sigma}\right)\bar{Z}_{\eta i\sigma} + \sum_{\sigma'}^{\alpha,\beta}\sum_{j}^{occ}\sum_{\eta,\tau}\bar{Z}_{\eta j\sigma'}\sum_{\nu} Q_{\mu\nu\sigma}\frac{\partial F_{\eta\tau\sigma'}}{\partial C_{\nu i\sigma}} C_{\tau j\sigma'} = 0 \tag{S22}$$

First we find that the second term in eq. (S22) vanishes due to the projection to the unoccupied space.

$$\sum_{\sigma'}^{\alpha,\beta}\sum_{k,j}^{occ}\sum_{\nu} Q_{\mu\nu\sigma}\frac{\partial S_{kj\sigma'}}{\partial C_{\nu i\sigma}}\bar{W}^{C}_{kj\sigma'} = 2\sum_{j}^{occ}\sum_{\nu,\tau} Q_{\mu\nu\sigma}S_{\nu\tau}C_{\tau j\sigma}\bar{W}^{C}_{ij\sigma} = 0 \tag{S23}$$

We expand the first term in eq. (S22) into three terms:

$$\begin{aligned}\sum_{\nu} Q_{\mu\nu\sigma}\frac{\partial G^{\alpha\to\beta}}{\partial C_{\nu i\sigma}} &= \sum_{j}^{occ}\sum_{\eta} X_{\eta j\alpha}\sum_{k}^{occ}\sum_{\tau}\sum_{\nu} Q_{\mu\nu\sigma}\frac{\partial\left[F_{\eta\tau\beta}\delta_{jk} - F_{jk\alpha}S_{\eta\tau}\right]}{\partial C_{\nu i\sigma}}X_{\tau k\alpha}\\ &+ \sum_{j}^{occ}\sum_{\eta,\tau} X_{\eta j\alpha}\sum_{\nu} Q_{\mu\nu\sigma}\frac{\partial C_{\tau j\alpha}K^{SF}_{\eta\tau}[\boldsymbol{D}^{X\alpha\to\beta}]}{\partial C_{\nu i\sigma}} - \delta_{\sigma\beta}\sum_{\nu} Q_{\mu\nu\sigma}\sum_{j}^{occ}\bar{W}^{X}_{i\beta j\alpha}\sum_{\tau} S_{\nu\tau}X_{\tau j\alpha}\end{aligned} \tag{S24}$$

The first term in eq. (S24) is very similar to the eq. (S15):

$$\begin{aligned}&\sum_{j}^{occ}\sum_{\eta} X_{\eta j\alpha}\sum_{k}^{occ}\sum_{\tau}\sum_{\nu} Q_{\mu\nu\sigma}\frac{\partial\left[F_{\eta\tau\beta}\delta_{jk} - F_{jk\alpha}S_{\eta\tau}\right]}{\partial C_{\nu i\sigma}}X_{\tau k\alpha}\\ &= \sum_{\sigma}\sum_{\eta,\tau}\sum_{\nu} Q_{\mu\nu\sigma}\frac{\partial F_{\eta\tau\sigma'}}{\partial C_{\nu i\sigma}}T_{\eta\tau\sigma'} - 2\delta_{\sigma\alpha}\sum_{k}^{occ}\sum_{\eta} X_{\eta i\alpha}\sum_{\tau,\nu} Q_{\mu\nu\sigma}F_{\nu\lambda\alpha}C_{\lambda k\alpha}S_{\eta\tau}X_{\tau k\alpha}\\ &= \sum_{\nu} Q_{\mu\nu\sigma}H_{\nu i\sigma}[\boldsymbol{T}]\end{aligned} \tag{S25}$$

The second term in eq. (S24) can be expanded as:

$$\sum_{j}^{occ}\sum_{\eta,\tau} X_{\eta j\alpha}\sum_{\nu} Q_{\mu\nu\sigma}\frac{\partial C_{\tau j\alpha}K^{SF}_{\eta\tau}[\boldsymbol{D}^{X\alpha\to\beta}]}{\partial C_{\nu i\sigma}} = 2\delta_{\alpha\sigma}\sum_{\eta,\nu} X_{\eta i\alpha}K^{SF}_{\eta\nu}[\boldsymbol{D}^{X\alpha\to\beta}]Q_{\mu\nu\sigma} + 2\sum_{\nu} Q_{\mu\nu\sigma}g^{SF}_{\nu i\sigma} \tag{S26}$$

The last term in eq. (S22) simply becomes:

$$\sum_{\sigma'}^{\alpha,\beta}\sum_{j}^{occ}\sum_{\eta,\tau}\bar{Z}_{\eta j\sigma'}\sum_{\nu} Q_{\mu\nu\sigma}\frac{\partial F_{\eta\tau\sigma'}}{\partial C_{\nu i\sigma}}C_{\tau j\sigma'} = \sum_{\sigma'}^{\alpha,\beta}\sum_{\nu} Q_{\mu\nu\sigma}\sum_{\eta,\tau}\frac{\partial F_{\eta\tau\sigma'}}{\partial C_{\nu i\sigma}}\sum_{j}^{occ}\bar{Z}_{\eta j\sigma'}C_{\tau j\sigma'} = \sum_{\sigma'}^{\alpha,\beta}\sum_{\nu} Q_{\mu\nu\sigma}H_{\nu i\sigma}[\boldsymbol{D}^{Z}] \tag{S27}$$

Thus, we find the Sternheimer Z-vector equation system.

$$\begin{aligned}
&\sum_{\eta,\nu} Q_{\mu\nu\sigma}\left(F_{\eta\nu\sigma} - S_{\eta\nu}\epsilon_{i\sigma}\right)\bar{Z}_{\eta i\sigma} + \sum_{\sigma'}^{\alpha,\beta}\sum_{\nu} Q_{\mu\nu\sigma} H_{\nu i\sigma}[\boldsymbol{D}^{Z}] \\
&\qquad = -\sum_{\nu} Q_{\mu\nu\sigma} H_{\nu i\sigma}[\boldsymbol{T}] - 2\delta_{\alpha\sigma}\sum_{\eta,\nu} X_{\eta i\alpha} K^{SF}_{\eta\nu}[\boldsymbol{D}^{X\alpha\to\beta}] Q_{\mu\nu\sigma} - 2\sum_{\nu} Q_{\mu\nu\sigma} g^{SF}_{\nu i\sigma} \\
&\qquad + 2\delta_{\sigma\beta}\sum_{\nu} Q_{\mu\nu\sigma}\sum_{j}^{occ} K^{SF}_{i\beta j\alpha}[\boldsymbol{D}^{X\alpha\to\beta}]\sum_{\tau} S_{\nu\tau} X_{\tau j\alpha}
\end{aligned} \tag{S28}$$

Optimized parameters

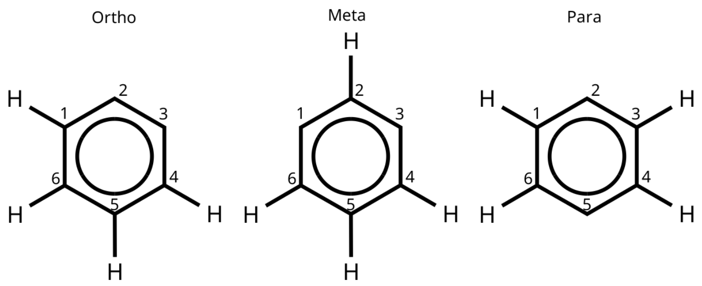

Figure 1: Scheme with numbering of ortho, meta and para benzynes.

Table S1: Structural parameters of two states of ortho-Benzyne. Bond lengths in Å and angles in degrees. DZVP basis set

	$^1A_1$							$^3B_2$						
	LDA	PBE	PBE0	PBE50	CCSD[a]	50-50[b]	LDA[c]	LDA	PBE	PBE0	PBE50	CCSD[a]	50-50[b]	LDA[c]
							Bonds							
C_1H_1	1.097	1.083	1.078	1.077	1.085	1.075	-	1.103	1.097	1.089	1.081	1.082	1.078	1.098
C_6H_6	1.102	1.090	1.084	1.080	1.081	1.078	-	1.101	1.094	1.089	1.079	1.081	1.077	1.094
C_1C_2	1.391	1.373	1.371	1.375	1.412	1.374	-	1.386	1.379	1.367	1.366	1.377	1.365	1.366
C_2C_3	1.273	1.259	1.253	1.245	1.335	1.241	-	1.405	1.383	1.382	1.386	1.395	1.385	1.385
C_4C_5	1.417	1.410	1.398	1.393	1.449	1.395	-	1.413	1.411	1.400	1.390	1.402	1.394	1.395
C_5C_6	1.410	1.408	1.401	1.395	1.363	1.398	-	1.401	1.402	1.392	1.380	1.390	1.381	1.383
							Angles							
$C_1C_2C_3$	126.4	126.6	126.6	126.6	120.5	126.8	-	120.5	121.0	121.3	120.8	121.0	121.1	120.7
$C_3C_4C_5$	110.9	111.4	111.2	110.8	120.5	110.9	-	119.3	119.0	118.3	118.7	118.5	118.4	119.3
$H_1C_1C_2$	127.1	126.1	125.8	126.9	121.9	126.7	-	120.1	121.3	121.0	120.6	120.7	120.8	120.2
$C_4C_5H_5$	118.1	118.1	118.1	118.3	119.8	118.9	-	119.4	119.9	119.5	119.3	119.5	119.4	119.6

[a]SF-CCSD/cc-pVTZ; [b]collinear SF-TDDFT/50-50/6-311G(d);[1] [c]noncollinear SF-TDDFT/LDA/cc-pVTZ.[2]

Table S2: Structure parameters of two states of ortho-Benzyne. Bond lengths in Å and angles in degrees. TZVP basis set

	$^1A_1$						$^3B_2$					
	PBE	PBE0	PBE50	CCSD[a]	50-50[b]	LDA[c]	PBE	PBE0	PBE50	CCSD[a]	50-50[b]	LDA[c]
						Bonds						
C_1H_1	1.082	1.078	1.078	1.085	1.075	-	1.097	1.087	1.081	1.082	1.078	1.098
C_6H_6	1.092	1.084	1.081	1.081	1.078	-	1.095	1.090	1.080	1.081	1.077	1.094
C_1C_2	1.375	1.371	1.376	1.412	1.374	-	1.378	1.365	1.367	1.377	1.365	1.366
C_2C_3	1.254	1.249	1.244	1.335	1.241	-	1.382	1.382	1.386	1.395	1.385	1.385
C_4C_5	1.411	1.399	1.393	1.449	1.395	-	1.409	1.394	1.392	1.402	1.394	1.395
C_5C_6	1.407	1.400	1.394	1.363	1.398	-	1.401	1.391	1.380	1.390	1.381	1.383
						Angles						
$C_1C_2C_3$	126.6	126.6	126.6	120.5	126.8	-	121.1	121.4	120.9	121.0	121.1	120.7
$C_3C_4C_5$	111.4	111.2	110.9	120.5	110.9	-	118.9	118.0	118.7	118.5	118.4	119.3
$H_1C_1C_2$	126.1	125.9	126.9	121.9	126.7	-	121.2	120.6	120.6	120.7	120.8	120.2
$C_4C_5H_5$	118.1	118.1	118.6	119.8	118.9	-	119.8	119.2	119.3	119.5	119.4	119.6

[a]SF-CCSD/cc-pVTZ; [b]collinear SF-TDDFT/50-50/6-311G(d);[1] [c]noncollinear SF-TDDFT/LDA/cc-pVTZ.[2]

Table S3: Structure parameters of two states of meta-Benzyne. Bond lengths in Å and angles in degrees. DZVP basis set

	$^1A_1$							$^3B_2$						
	LDA	PBE	PBE0	PBE50	CCSD[a]	50-50[b]	LDA[c]	LDA	PBE	PBE0	PBE50	CCSD[a]	50-50[b]	LDA[c]
							Bonds							
C_2H_2	1.099	1.090	1.079	1.062	1.075	1.073	1.096	1.106	1.093	1.087	1.084	1.081	1.078	1.098
C_4H_4	1.100	1.094	1.085	1.085	1.080	1.076	1.092	1.103	1.091	1.087	1.077	1.080	1.076	1.094
C_5H_5	1.103	1.085	1.078	1.092	1.084	1.080	1.096	1.099	1.100	1.089	1.080	1.082	1.078	1.096
C_1C_2	1.372	1.366	1.359	1.379	1.362	1.353	1.354	1.391	1.373	1.374	1.368	1.376	1.370	1.370
C_3C_4	1.380	1.374	1.366	1.334	1.368	1.362	1.362	1.387	1.380	1.373	1.366	1.373	1.366	1.366
C_4C_5	1.409	1.399	1.388	1.414	1.392	1.389	1.391	1.412	1.402	1.399	1.388	1.396	1.392	1.395
							Angles							
$C_1C_2C_3$	92.0	95.6	97.6	112.7	98.3	96.3	91.6	115.5	114.6	114.3	115.0	114.9	114.8	115.2
$C_2C_3C_4$	141.7	139.1	137.4	126.9	136.8	138.5	142.0	124.7	125.4	125.5	125.0	124.9	125.1	125.1
$C_3C_4C_5$	116.0	115.8	116.4	116.9	116.8	116.4	116.0	116.6	116.6	116.9	116.7	116.8	116.7	116.4
$C_3C_4H_4$	121.3	122.6	122.0	124.7	120.6	121.1	121.7	122.7	122.6	122.4	122.4	122.3	122.4	123.0

[a]SF-CCSD/cc-pVTZ; [b]collinear SF-TDDFT/50-50/6-311G(d);[1] [c]noncollinear SF-TDDFT/LDA/cc-pVTZ.[2]

Table S4: Structural parameters of two states of meta-Benzyne. Bond lengths in Å and angles in degrees. TZVP basis set

	$^1A_1$						$^3B_2$					
	PBE	PBE0	PBE50	CCSD[a]	50-50[b]	LDA[c]	PBE	PBE0	PBE50	CCSD[a]	50-50[b]	LDA[c]
						Bonds						
C_2H_2	1.086	1.078	1.078	1.075	1.073	1.096	1.093	1.088	1.083	1.081	1.078	1.098
C_4H_4	1.095	1.086	1.075	1.080	1.076	1.092	1.091	1.088	1.077	1.080	1.076	1.094
C_5H_5	1.087	1.085	1.079	1.084	1.080	1.096	1.102	1.090	1.079	1.082	1.078	1.096
C_1C_2	1.369	1.360	1.333	1.362	1.353	1.354	1.373	1.373	1.369	1.376	1.370	1.370
C_3C_4	1.370	1.365	1.366	1.368	1.362	1.362	1.379	1.372	1.368	1.373	1.366	1.366
C_4C_5	1.401	1.391	1.397	1.392	1.389	1.391	1.404	1.398	1.390	1.396	1.392	1.395
						Angles						
$C_1C_2C_3$	94.9	96.4	69.6	98.3	96.3	91.6	114.6	114.0	114.7	114.9	114.8	115.2
$C_2C_3C_4$	139.5	138.3	162.2	136.8	138.5	142.0	125.5	125.6	125.2	124.9	125.1	125.1
$C_3C_4C_5$	115.9	116.3	106.8	116.8	116.4	116.0	116.5	117.0	116.7	116.8	116.7	116.4
$C_3C_4H_4$	122.4	122.1	128.0	120.6	121.1	121.7	122.8	122.4	122.5	122.3	122.4	123.0

[a]SF-CCSD/cc-pVTZ; [b]collinear SF-TDDFT/50-50/6-311G(d);[1] [c]noncollinear SF-TDDFT/LDA/cc-pVTZ.[2]

Table S5: Structural parameters of two states of para-Benzyne. Bond lengths in Å and angles in degrees. DZVP basis set

	$^1A_1$							$^3B_2$						
	LDA	PBE	PBE0	PBE50	CCSD[a]	50-50[b]	LDA[c]	LDA	PBE	PBE0	PBE50	CCSD[a]	50-50[b]	LDA[c]
	Bonds													
C_1H_1	1.100	1.091	1.089	1.077	1.080	1.076	1.093	1.101	1.087	1.084	1.079	1.081	1.078	1.096
C_1C_2	1.365	1.362	1.363	1.353	1.369	1.355	1.343	1.386	1.372	1.368	1.367	1.378	1.367	1.366
C_3C_4	1.458	1.455	1.422	1.417	1.422	1.419	1.445	1.416	1.405	1.399	1.395	1.403	1.396	1.400
	Angles													
$C_1C_2C_3$	124.4	125.0	126.0	125.0	124.6	124.9	124.8	127.2	128.0	126.9	126.5	126.6	126.9	127.6
$C_2C_3C_4$	117.8	117.5	117.0	117.5	117.7	117.5	117.6	116.4	116.0	116.5	116.7	116.7	116.5	116.2
$H_6C_6C_1$	116.6	118.5	119.6	119.0	118.9	118.7	116.1	120.7	120.5	120.5	120.7	121.2	121.1	121.1

[a]SF-CCSD/cc-pVTZ; [b]collinear SF-TDDFT/50-50/6-311G(d);[1] [c]noncollinear SF-TDDFT/LDA/cc-pVTZ.[2]

Table S6: Structural parameters of two states of para-Benzyne. Bond lengths in Å and angles in degrees. TZVP basis set

	$^1A_1$						$^3B_2$					
	PBE	PBE0	PBE50	CCSD[a]	50-50[b]	LDA[c]	PBE	PBE0	PBE50	CCSD[a]	50-50[b]	LDA[c]
	Bonds											
C_1H_1	1.092	1.091	1.079	1.080	1.076	1.093	1.087	1.087	1.079	1.081	1.078	1.096
C_1C_2	1.361	1.365	1.354	1.369	1.355	1.343	1.371	1.371	1.364	1.378	1.367	1.366
C_3C_4	1.453	1.420	1.420	1.422	1.419	1.445	1.407	1.398	1.392	1.403	1.396	1.400
	Angles											
$C_1C_2C_3$	125.1	125.7	124.8	124.6	124.9	124.8	128.0	127.3	127.2	126.6	126.9	127.6
$C_2C_3C_4$	117.4	117.1	117.6	117.7	117.5	117.6	116.0	116.3	116.4	116.7	116.5	116.2
$H_6C_6C_1$	118.6	119.6	118.9	118.9	118.7	116.1	120.5	120.6	120.9	121.2	121.1	121.1

[a]SF-CCSD/cc-pVTZ; [b]collinear SF-TDDFT/50-50/6-311G(d);[1] [c]noncollinear SF-TDDFT/LDA/cc-pVTZ.[2]

Table S7: Optimized structural parameters. Bond lengths in Å and angles in degrees. DZVP basis set

	CH_2				NH_2^+			
	$^3B_1$	$^1A_1$	$^1B_1$	2^1A_1	$^3B_1$	$^1A_1$	$^1B_1$	2^1A_1
				Bonds				
LDA	1.091	1.127	1.088	1.082	1.045	1.068	1.049	1.053
PBE	1.076	1.098	1.078	1.074	1.031	1.056	1.038	1.048
PBE0	1.076	1.110	1.073	1.067	1.027	1.032	1.027	1.034
PBE50	1.072	1.110	1.069	1.060	1.027	1.032	1.025	1.025
CISD[a]	1.078	1.109	1.075	1.068	1.030	1.046	1.029	1.032
				Angles				
LDA	132.4	102.1	142.1	165.2	150.3	109.9	159.8	173.9
PBE	133.4	105.3	140.0	163.2	152.7	105.9	156.6	179.9
PBE0	133.5	102.2	137.1	166.6	151.1	139.8	152.9	174.6
PBE50	134.1	124.0	143.2	172.6	150.5	119.0	162.4	178.8
CISD[a]	133.3	101.9	141.6	170.1	150.9	108.0	161.5	180.0
	SiH_2				PH_2^+			
	$^1A_1$	$^3B_1$	$^1B_1$	2^1A_1	$^1A_1$	$^3B_1$	$^1B_1$	2^1A_1
				Bonds				
LDA	1.550	1.501	1.511	1.481	1.453	1.429	1.448	1.440
PBE	1.542	1.493	1.508	1.476	1.455	1.430	1.426	1.777
PBE0	1.522	1.476	1.488	1.462	1.406	1.418	1.431	1.423
PBE50	1.513	1.476	1.479	1.450	1.422	1.412	1.423	1.407
CISD[a]	1.515	1.477	1.483	1.457	1.418	1.406	1.419	1.412
				Angles				
LDA	89.8	116.8	119.4	154.2	90.7	120.4	123.0	155.0
PBE	91.4	116.5	121.0	151.0	91.4	117.6	126.2	180.0
PBE0	91.7	118.3	122.0	153.1	121.8	119.3	125.3	154.3
PBE50	91.7	118.0	121.2	160.0	92.1	121.0	123.9	160.6
CISD[a]	92.7	118.3	122.7	162.3	93.1	121.8	124.8	159.6

[a]CISD/TZ2P(f,d);[3–5] FCI/TZ2P instead of CISD for CH_2.[6]

Table S8: Optimized structural parameters. Bond lengths in Å and angles in degrees. TZVP basis set

	CH_2				NH_2^+			
	$^3B_1$	$^1A_1$	$^1B_1$	2^1A_1	$^3B_1$	$^1A_1$	$^1B_1$	2^1A_1
				Bonds				
PBE	1.078	1.109	1.059	1.076	1.037	1.046	1.053	1.048
PBE0	1.078	1.099	1.076	1.067	1.029	1.050	1.032	1.037
PBE50	1.074	1.110	1.070	1.060	1.029	1.047	1.029	1.028
CISD[a]	1.078	1.109	1.075	1.068	1.030	1.046	1.029	1.032
				Angles				
PBE	133.3	119.0	141.8	162.6	153.9	108.0	157.9	179.9
PBE0	134.3	104.9	140.2	166.8	151.0	110.9	152.9	174.8
PBE50	134.3	124.0	144.6	173.2	150.6	118.8	159.6	178.3
CISD[a]	133.3	101.9	141.6	170.1	150.9	108.0	161.5	180.0
	SiH_2				PH_2^+			
	$^1A_1$	$^3B_1$	$^1B_1$	2^1A_1	$^1A_1$	$^3B_1$	$^1B_1$	2^1A_1
				Bonds				
PBE	1.550	1.492	1.507	1.477	1.440	1.430	1.440	1.613
PBE0	1.533	1.477	1.493	1.467	1.433	1.418	1.437	1.424
PBE50	1.518	1.477	1.482	1.455	1.423	1.411	1.422	1.407
CISD[a]	1.515	1.477	1.483	1.457	1.418	1.406	1.419	1.412
				Angles				
PBE	92.9	117.9	121.3	151.4	91.6	117.6	123.9	180.0
PBE0	91.2	118.3	123.2	154.5	91.3	119.4	125.7	154.1
PBE50	91.4	118.2	121.8	160.8	92.3	121.0	124.1	160.6
CISD[a]	92.7	118.3	122.7	162.3	93.1	121.8	124.8	159.6

[a]CISD/TZ2P(f,d);[3–5] FCI/TZ2P instead of CISD for CH_2.[6]

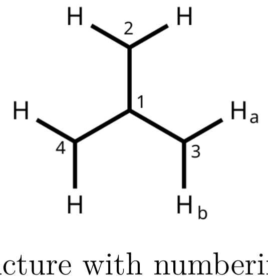

Figure 2: Structure with numbering of TMMM.

Table S9: Structural parameters of TMM states. Bond lengths in Å and angles in degrees. C_{2v} symmetry labels are used. DZVP basis set

	$^3B_1$					$^1B_1$				
	LDA	PBE	PBE0	PBE50	5050[a]	LDA	PBE	PBE0	PBE50	5050[a]
					Bonds					
C_2H	1.0977	1.0894	1.0807	1.0761	1.0755	1.0755	1.0755	1.0756	1.0755	1.0766
C_3H	1.0971	1.0889	1.0808	1.0760	1.0755	1.0755	1.0755	1.0756	1.0755	1.0749
C_1C_2	1.4180	1.4121	1.4055	1.4018	1.4021	1.4021	1.4021	1.4019	1.4021	1.4818
C_1C_3	1.4174	1.4115	1.4057	1.4018	1.4021	1.4021	1.4021	1.4020	1.4021	1.3743
					Angles					
C_1C_2H	120.92	120.74	120.95	121.18	121.16	121.16	121.16	121.17	121.16	120.88
C_1C_3Ha	120.89	120.76	120.95	121.18	121.16	121.16	121.16	121.17	121.16	121.66
C_1C_3Hb	120.95	120.73	120.96	121.18	121.16	121.16	121.16	121.17	121.16	120.83
$C_2C_1C_3$	120.00	120.00	120.01	120.00	120.00	120.00	120.00	120.00	120.00	119.21
	$^1A_1$					2^1A_1				
	LDA	PBE	PBE0	PBE50	5050[a]	LDA	PBE	PBE0	PBE50	5050[a]
					Bonds					
C_2H	1.0980	1.0853	1.0790	1.0752	1.0773	1.0971	1.0877	1.0817	1.0742	1.0749
C_3H	1.0964	1.0861	1.0817	1.0762	1.0744	1.0965	1.0877	1.0813	1.0748	1.0749
C_1C_2	1.3760	1.3599	1.4752	1.4315	1.3384	1.4181	1.4162	1.3957	1.3876	1.3925
C_1C_3	1.4450	1.4570	1.3381	1.3395	1.4526	1.4177	1.4162	1.3981	1.3888	1.3925
					Angles					
C_1C_2H	121.08	120.83	119.93	120.21	121.54	120.91	120.90	121.22	120.93	121.20
C_1C_3Ha	120.32	119.90	120.76	121.40	120.42	120.87	120.90	121.13	120.90	121.20
C_1C_3Hb	121.04	120.69	121.64	121.42	121.24	120.93	120.90	121.23	120.90	121.20
$C_2C_1C_3$	120.81	121.28	121.50	121.54	120.90	119.99	120.00	120.05	120.03	120.00

[a]collinear SF-TDDFT/50-50/6-311G(d).[1]

Table S10: Structural parameters of TMM states. Bond lengths in Å and angles in degrees. C_{2v} symmetry labels are used. TZVP basis set

	$^3B_1$				$^1B_1$			
	PBE	PBE0	PBE50	5050[a]	PBE	PBE0	PBE50	5050[a]
				Bonds				
C_2H	1.0904	1.0817	1.0750	1.0755	1.0755	1.0755	1.0755	1.0766
C_3H	1.0904	1.0817	1.0753	1.0755	1.0755	1.0755	1.0755	1.0749
C_1C_2	1.4148	1.4040	1.3971	1.4021	1.4022	1.4020	1.4021	1.4818
C_1C_3	1.4132	1.4042	1.3976	1.4021	1.4022	1.4022	1.4021	1.3743
				Angles				
C_1C_2H	121.09	121.03	120.91	121.16	121.16	121.16	121.16	120.88
C_1C_3Ha	120.99	120.97	120.98	121.16	121.16	121.16	121.16	121.66
C_1C_3Hb	120.87	121.00	120.92	121.16	121.15	121.16	121.16	120.83
$C_2C_1C_3$	119.86	120.01	120.01	120.00	119.99	120.00	120.00	119.21
	$^1A_1$				2^1A_1			
	PBE	PBE0	PBE50	5050[a]	PBE	PBE0	PBE50	5050[a]
				Bonds				
C_2H	1.0888	1.0799	1.0758	1.0773	1.0880	1.0817	1.0767	1.0749
C_3H	1.0891	1.0817	1.0753	1.0744	1.0880	1.0818	1.0770	1.0749
C_1C_2	1.4638	1.4777	1.4600	1.3384	1.4158	1.4018	1.3890	1.3925
C_1C_3	1.3570	1.3394	1.3351	1.4526	1.4160	1.4018	1.3882	1.3925
				Angles				
C_1C_2H	120.36	118.80	120.09	121.54	120.91	121.03	120.89	121.20
C_1C_3Ha	121.04	121.20	121.36	120.42	120.90	121.04	120.98	121.20
C_1C_3Hb	121.12	121.51	121.55	121.24	120.91	121.03	120.90	121.20
$C_2C_1C_3$	121.17	121.17	120.93	120.90	120.01	120.00	119.98	120.00

[a]collinear SF-TDDFT/50-50/6-311G(d).[1]

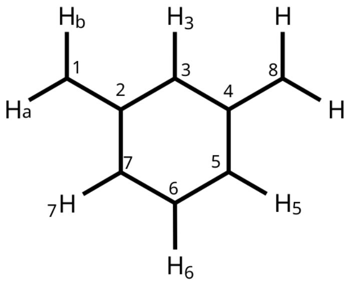

Figure 3: Structure with numbering of meta-xylylene.

Table S11: Structural parameters of meta-xylylene states. Bond lengths in Å and angles in degrees. C_{2v} symmetry labels are used. DZVP basis set

	$^3B_2$					$^1A_1$					$^1B_2$				
	LDA	PBE	PBE0	PBE50	CCSD[a]	LDA	PBE	PBE0	PBE50	CCSD[a]	LDA	PBE	PBE0	PBE50	CCSD[a]
								Bonds							
C_1H_a	1.187	1.090	1.083	1.076	1.087	1.188	1.089	1.083	1.078	1.086	1.186	1.090	1.081	1.074	1.088
C_3H_3	1.103	1.093	1.084	1.077	1.090	1.103	1.088	1.082	1.076	1.091	1.103	1.096	1.084	1.077	1.089
C_5H_5	1.124	1.091	1.084	1.077	1.089	1.124	1.090	1.084	1.077	1.089	1.122	1.093	1.083	1.078	1.088
C_6H_6	1.064	1.091	1.083	1.077	1.089	1.065	1.090	1.083	1.079	1.089	1.063	1.092	1.082	1.078	1.090
C_1C_2	1.407	1.409	1.407	1.388	1.402	1.383	1.372	1.348	1.343	1.452	1.424	1.433	1.447	1.430	1.345
C_2C_3	1.430	1.418	1.406	1.402	1.419	1.443	1.437	1.441	1.428	1.400	1.424	1.406	1.396	1.392	1.445
C_4C_5	1.445	1.431	1.418	1.414	1.433	1.466	1.463	1.472	1.463	1.402	1.432	1.418	1.397	1.395	1.490
C_5C_6	1.408	1.393	1.383	1.374	1.392	1.407	1.392	1.381	1.371	1.389	1.408	1.392	1.385	1.376	1.384
								Angles							
$C_2C_1H_a$	119.6	121.0	121.0	121.0	121.3	120.0	121.5	121.9	122.0	121.0	119.0	120.8	120.7	121.0	121.5
$C_2C_1H_b$	123.3	121.1	121.1	121.2	121.3	123.0	121.2	121.4	121.5	120.9	123.8	120.8	120.8	121.0	121.7
$C_4C_5H_5$	119.2	119.0	119.0	119.1	119.0	118.8	118.0	117.9	118.4	119.4	119.4	119.5	119.6	119.5	118.0
$C_1C_2C_3$	120.5	121.2	121.3	121.4	121.3	121.0	121.8	122.0	122.0	121.0	119.8	120.8	121.0	121.3	122.2
$C_2C_3C_4$	124.1	122.1	122.3	122.3	122.1	124.3	122.9	122.9	122.7	121.7	123.9	121.3	121.8	122.4	122.3
$C_3C_4C_5$	117.4	117.8	117.7	117.6	117.8	116.8	116.7	116.4	116.6	118.5	117.7	118.6	118.5	117.8	116.8
$C_4C_5C_6$	118.0	120.7	120.7	120.7	120.7	118.3	121.0	121.0	120.9	120.5	117.8	120.4	120.5	120.7	121.0

[a] CCSD/6-31G(d) for triplets and EOM-SF-CCSD/6-31G(d) for singlets.[7]

Table S12: Structural parameters of meta-xylylene states. Bond lengths in Å and angles in degrees. C_{2v} symmetry labels are used. TZVP basis set

	$^3B_2$				$^1A_1$				$^1B_2$			
	PBE	PBE0	PBE50	CCSD[a]	PBE	PBE0	PBE50	CCSD[b]	PBE	PBE0	PBE50	CCSD[b]
						Bonds						
C_1H_a	1.091	1.084	1.076	1.087	1.091	1.083	1.079	1.086	1.090	1.082	1.076	1.088
C_3H_3	1.094	1.086	1.079	1.090	1.092	1.084	1.077	1.091	1.094	1.086	1.079	1.089
C_5H_5	1.092	1.084	1.077	1.089	1.092	1.084	1.076	1.089	1.092	1.083	1.078	1.088
C_6H_6	1.091	1.084	1.077	1.089	1.092	1.084	1.076	1.089	1.091	1.086	1.077	1.090
C_1C_2	1.409	1.408	1.389	1.402	1.369	1.350	1.342	1.452	1.439	1.447	1.431	1.345
C_2C_3	1.418	1.405	1.403	1.419	1.440	1.439	1.427	1.400	1.407	1.392	1.390	1.445
C_4C_5	1.431	1.418	1.415	1.433	1.467	1.469	1.463	1.402	1.416	1.402	1.394	1.490
C_5C_6	1.393	1.382	1.374	1.392	1.391	1.381	1.370	1.389	1.394	1.382	1.377	1.384
						Angles						
$C_2C_1H_a$	121.0	121.1	120.9	121.3	121.5	121.8	121.5	121.0	120.7	120.7	120.9	121.5
$C_2C_1H_b$	121.1	121.2	121.1	121.3	121.1	121.1	121.2	120.9	120.6	120.7	120.8	121.7
$C_4C_5H_5$	118.9	119.0	119.1	119.0	118.1	118.2	118.6	119.4	119.4	119.3	119.5	118.0
$C_1C_2C_3$	121.3	121.3	121.4	121.3	121.9	121.8	121.9	121.0	121.0	121.1	121.3	122.2
$C_2C_3C_4$	122.2	122.3	122.3	122.1	122.8	122.5	122.3	121.7	121.7	122.0	122.2	122.3
$C_3C_4C_5$	117.8	117.7	117.5	117.8	116.7	116.6	116.9	118.5	118.4	118.2	118.0	116.8
$C_4C_5C_6$	120.7	120.7	120.7	120.7	121.0	121.1	121.0	120.5	120.5	120.6	120.5	121.0

[a]CCSD/6-31G(d) for triplets and EOM-SF-CCSD/6-31G(d) for singlets.[7]

References

(1) Slipchenko, L. V.; Krylov, A. I. Singlet-triplet gaps in diradicals by the spin-flip approach: A benchmark study. *The Journal of Chemical Physics* **2002**, *117*, 4694–4708.

(2) Bernard, Y. A.; Shao, Y.; Krylov, A. I. General formulation of spin-flip time-dependent density functional theory using non-collinear kernels: Theory, implementation, and benchmarks. *The Journal of Chemical Physics* **2012**, *136*, 204103.

(3) Stephens, J. C.; Yamaguchi, Y.; Sherrill, C. D.; Schaefer, H. F. X 3B1, a 1A1, b 1B1, and b 1 Electronic States of. *The Journal of Physical Chemistry A* **1998**, *102*, 3999–4006.

(4) Yamaguchi, Y.; Van Huis, T. J.; Sherrill, C. D.; Schaefer III, H. F. The X 1A1, a 3B1, a 1B1, and B 1A1 electronic states of SiH2. *Theoretical Chemistry Accounts* **1997**, *97*, 341–349.

(5) Van Huis, T. J.; Yamaguchi, Y.; Sherrill, C. D.; Schaefer, H. F. X 1A1, a 3B1, A 1B1, and B 1A1 Electronic States of. *The Journal of Physical Chemistry A* **1997**, *101*, 6955–6963.

(6) Sherrill, C. D.; Leininger, M. L.; Van Huis, T. J.; Schaefer, I., Henry F. Structures and vibrational frequencies in the full configuration interaction limit: Predictions for four electronic states of methylene using a triple-zeta plus double polarization (TZ2P) basis. *The Journal of Chemical Physics* **1998**, *108*, 1040–1049.

(7) Wang, T.; Krylov, A. I. The effect of substituents on electronic states' ordering in meta-xylylene diradicals: Qualitative insights from quantitative studies. *The Journal of Chemical Physics* **2005**, *123*, 104304.
